# Dependencies in heterogeneous, lineage plastic patient–derived prostate cancer organoids revealed through integrated single–cell multiomics and CRISPR screening

**DOI:** 10.64898/2026.05.07.723570

**Published:** 2026-05-08

**Authors:** Samir Zaidi, Maren Büttner, Weiran Feng, Caitlin Baxter, D. Henry Kates, Tiago Paiva Prudente, Diana Zakharova, Sanjoy Mehta, Subhiksha Nandakumar, Chen Khuan Wong, Hojin Lee, Pierre–Jacques Hamard, Wenfei Kang, Jungmin Choi, Ronan Chaligné, Robert Cohen, Yu Chen, Ari Firestone, Zhenghao Chen, Charles L. Sawyers

**Affiliations:** 1. Department of Medicine, Yale School of Medicine, New Haven, CT 06510, USA.; 2. Center of Molecular and Cellular Oncology, Yale Cancer Center, New Haven, CT 06510, USA.; 3. Calico Life Sciences LLC, South San Francisco, CA USA; 4. Cancer Epigenetics Institute, Nuclear Dynamics & Cancer Program Fox Chase Cancer Center, Philadelphia, PA 19111, USA.; 5. Human Oncology and Pathogenesis Program, Memorial Sloan Kettering Cancer Center, New York, NY 10065, USA.; 6. Genome Editing and Screening Core, Memorial Sloan Kettering Cancer Center, New York, NY 10065, USA.; 7. Department of Epidemiology and Biostatistics, Memorial Sloan Kettering Cancer Center, New York, NY 10065, USA.; 8. Department of Biomedical Sciences, Korea University College of Medicine, Seoul, Korea.; 9. Epigenetic Research Innovation Lab, Memorial Sloan Kettering Cancer Center, New York, NY 10065, USA.; 10. Molecular Cytology Core Facility, Memorial Sloan Kettering Cancer Center, New York, NY 10065, USA.; 11. Single Cell Analytics Innovation Lab, Memorial Sloan Kettering Cancer Center, New York, NY 10065, USA.; 12. Department of Genitourinary Oncology, Memorial Sloan Kettering Cancer Center, New York, NY 10065, USA.; 13. Howard Hughes Medical Institute, Memorial Sloan Kettering Cancer Center, New York, NY 10065, USA.

## Abstract

Lineage plasticity and tumor heterogeneity limit the effectiveness of targeted therapies, yet the functional dependencies used to nominate therapeutic targets are often derived from homogeneous systems that fail to capture this complexity. Here, we establish a framework to resolve state–specific genetic vulnerabilities by integrating single–cell multiomics (RNA and ATAC) with pooled CRISPR–Cas9 screening across a large panel of patient–derived organoids (PDOs) from castrate–resistant prostate cancer (CRPC) and neuroendocrine prostate cancer (NEPC). We generate a single–cell multiome atlas spanning >190,000 cells across 22 PDOs, defining seven lineage states—including intermediate and plastic populations not resolved by bulk profiling—and demonstrate that these lineage programs robustly classify independent transcriptomic datasets from prostate cancer patient tumors. By systematically coupling this atlas to subtype–resolved CRISPR screens, we construct a functional dependency map linking cell state in heterogeneous 3D human tumor models. We show that intratumoral heterogeneity fundamentally reshapes the interpretation of gene essentiality, whereby gene–level depletion reflects the composite behavior of co–existing subpopulations, and identify a general principle in which resistant “limiting” populations disproportionately determine aggregate fitness effects. This framework reveals both canonical and previously unrecognized lineage–restricted dependencies within highly plastic tumor and NEPC states, including a therapeutically targetable dependency on the aryl hydrocarbon receptor (AHR) in a novel hybrid stem–like/ASCL1 population. Together, these data establish an extensive multi–dimensional prostate cancer resource, identify novel lineage–resolved biology, and provide a generalizable strategy for interpreting functional genomics in heterogeneous human tumors.

## Introduction

Tumor heterogeneity is a defining feature of advanced malignancies and a major driver of therapeutic resistance^1^. Despite extensive transcriptional and epigenetic profiling, the functional vulnerabilities of distinct tumor cell states—and how these vulnerabilities are shaped by their coexistence within heterogeneous tumors—remain poorly resolved^2–4^. A central challenge is that functional dependencies are typically inferred from bulk or homogeneous systems, implicitly assuming uniform dependency landscapes, despite growing evidence that intratumoral heterogeneity profoundly alters both the strength and interpretability of such signals^5^. This is particularly evident in cancers treated with targeted therapies, where selective pressure promotes adaptive diversification of cell states rather than durable eradication^6^. A key mechanism underlying this process is lineage plasticity, the ability of tumor cells to reversibly alter cellular identity in response to environmental or therapeutic stress, which is associated with poor clinical outcomes across multiple tumor types^7^.

Prostate cancer represents a paradigmatic model of therapy–induced lineage plasticity. Following treatment with androgen receptor pathway inhibitors (ARPIs), tumors frequently transition from androgen–dependent adenocarcinoma toward alternative lineage states, culminating in its most extreme form into neuroendocrine prostate cancer (NEPC), a highly aggressive and treatment–resistant disease^8, 9^. Recent profiling studies have broadly classified prostate cancer heterogeneity into androgen–dependent, WNT–driven, and neuroendocrine lineages, as well as a fourth lineage with AR–low or AR-negative states—often described as stem–like, poorly differentiated, or lineage–ambiguous^10^. However, the latter populations feature intermediate and plastic states, which exhibit substantial internal heterogeneity, often co–exist within tumors, and are poorly resolved by bulk analyses,^2^ leaving their regulatory programs and therapeutic vulnerabilities incompletely understood.

We and others have identified a distinct plastic AR–low state driven by JAK/STAT and FGFR signaling, that can be therapeutically modulated^8, 11^. Epigenetic regulators such as *EZH2* and *NSD2* have been implicated in maintaining AR–low and NEPC states, highlighting a potential path forward for lineage–directed therapies^12, 13,14^. Despite these advances, clinical translation remains limited, in part because current approaches fail to adequately resolve the diversity of plastic states or to match therapies to specific tumor subpopulations.

Several fundamental questions remain: (1) which transcription factors and druggable genes sustain heterogeneous plastic states, (2) can these dependencies be selectively targeted without inducing compensatory transitions, and (3) how does intratumoral heterogeneity shape functional dependency landscapes? Addressing these challenges has been difficult due to limitations of bulk profiling and the inability of most functional genomics platforms to directly link cell state with genetic dependency in the context of heterogeneous model systems. Additionally, homogenous models of AR–low and NEPC states are underrepresented in resources such as DepMap projects highlighting the need for more realistic models that faithfully recapitulate these clinically important lineages.

Patient–derived organoids (PDOs) preserve tumor heterogeneity and co–existing lineage states, making them well–suited to study lineage plasticity. Here, we address the limited availability of functional genomics resource across prostate cancer lineage states by integrating single–cell transcriptomic and chromatin accessibility profiling with pooled CRISPR–Cas9 screening across a large panel of human prostate cancer PDOs. We construct a single cell multiome atlas spanning 22 PDOs and define seven major lineage states, including intermediate and plastic populations obscured in bulk analyses. Leveraging this framework, we perform subtype–resolved CRISPR screens to map functional dependencies across lineage states. By linking gene depletion to transcriptional and chromatin states, we uncover state–specific vulnerabilities and demonstrate how intratumoral heterogeneity alters the interpretation of functional screens. Using this framework, we identify both established and previously unrecognized lineage–restricted dependencies, including actionable vulnerabilities within highly plastic states and a context–dependent dependency on the aryl hydrocarbon receptor (AHR) in prostate cancer. Together, these data establish a comprehensive multi–dimensional resource linking cell state, regulatory programs, and functional dependencies, and provide a generalizable framework for targeting lineage plasticity in advanced prostate cancer and other solid tumors.

## RESULTS

### Single Cell Multiome Analyses of PDO Atlas

To resolve cell heterogeneity and regulatory dynamics beyond the limits of bulk tumor profiling, we performed paired single–cell gene expression and chromatin accessibility (sc–multiome) sequencing on 22 PDOs generated from castrate–resistant prostate cancer (CRPC) and NEPC tumors. Seventeen of these PDOs had been previously classified by bulk RNA– and ATAC–sequencing into four major subtypes: AR (androgen–receptor), SCL (stem–cell–like), WNT (WNT signaling), and NEPC^10^. While this classification scheme has been informative, we sought to determine whether sc–multiome profiling could refine lineage definitions, uncover regulatory states not captured by gene expression alone, and quantify heterogeneity within and across PDOs.

**Figure 1.**
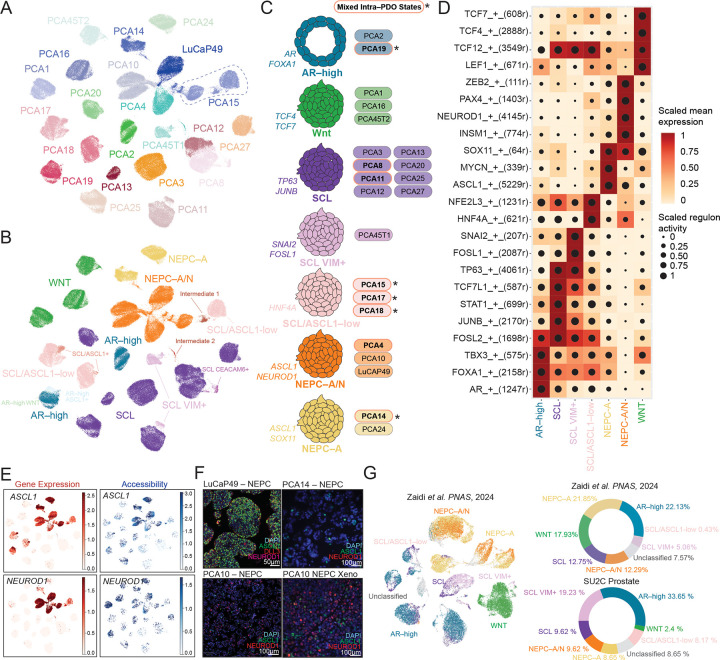
Single–cell multiome atlas of lineage states across prostate cancer organoids (PDOs). **(A)** UMAP projection of 22 PDO profiled by single–cell RNA and ATAC (sc–multiome) profiling, colored by PDO identity. Dotted line highlights PCA15 – an example of a PDO with multiple subpopulations. **(B)** UMAP colored by seven major lineage states, identifying AR–high, WNT, SCL, SCL VIM+, SCL/ASCL1–low, NEPC–A, NEPC–A/N, and multiple intermediate populations. **(C)** Schematic summarizing seven major lineage assignments, highlighting intra–PDO heterogeneity (asterisks and red bolded circle denote PDOs with mixed populations). **(D)** Scaled mean expression (color) and regulon activity (dot size) of differential lineage–defining transcription factors across subtypes using SCENIC+. **(E)** UMAP showing gene expression and chromatin accessibility for *ASCL1* and *NEUROD1*. **(F)** Immunofluorescence of neuroendocrine markers (ASCL1, NEUROD1, DAPI +/− DLL3) across representative NEPC PDOs. Scale bars, 50–100 μm. **(G)** UMAP of sc–RNA dataset from CRPC patient tumors [PMID: 38968122] labeled by PDO–defined lineage states using nearest template prediction (NTP) approach (left). Subtype composition in both sc–RNA–seq dataset and SU2C bulk tumors [PMID: 31061129] (right).

UMAP projection of 195,180 profiled cells revealed substantial heterogeneity across 22 PDOs, which largely segregated by sample (Fig 1A). Annotation by bulk–defined subtypes and cell cycle states were not primary drivers of this separation (Fig S1A and S1B). We therefore applied a multi–stage sub–clustering strategy to identify shared transcriptional and regulatory programs across PDOs. Briefly, we first performed PDO–specific Leiden clustering independently for RNA and ATAC modalities to avoid cross–sample averaging, followed by annotation using established lineage markers (e.g., *KLK3, TP63, NKD1, ASCL1,* and *NEUROD1*; Table S1). Subtypes were retained if their top 20 marker genes or peaks were differentially enriched relative to the remaining subtypes across the atlas (Fig S1C).

This approach resolved seven major subtypes (AR–high, SCL, SCL/VIM+, SCL/ASCL1–low, NEPC–A, NEPC–A/N, WNT) (Fig 1B and Fig 1C). These subtypes were largely independent of shared genomic alterations, except for WNT PDOs, which harbored alterations in Wnt pathway genes (Fig S1D). We also identified several minor and transitory populations (Fig 1B). To further refine the enhancer–driven gene–regulatory networks (GRNs) specific to individual PDOs (Fig S1E) and lineage subtypes (Fig 1D), we applied SCENIC+^15^ to integrate the regulatory basis of these states. These analyses identified subtype–specific regulatory programs consistent with prior functional and genetic observations and uncovered refined subclassifications within SCL and NEPC states. Consistent with the prior bulk–classification framework, the AR–high PDOs (PCA2, PCA19) showed increased *AR* and *FOXA1* activity, in line with their known sensitivity to AR pathway inhibitors (ARPI)^16^. Similarly, WNT PDOs (PCA1, PCA16, PCA45T2) showed high expression and regulon activity for *TCF7* and *TCF4*, consistent with canonical WNT signaling^10^ (Fig 1D).

Given the known plasticity of bulk–defined SCL and NEPC states^10^, we next leveraged sc–multiome profiling to resolve the heterogeneity within lineage groups. Notably, SCL organoids showed three distinct subtypes. SCL/ASCL1–low PDOs (PCA17, 18) lacked *AR* expression, showed high *HNF4A* activity, and exhibited increased *ASCL1* expression with accessibility profiles distinct from NEPC PDOs (Fig 1D). The SCL/VIM+ PDO (PCA45T1) displayed a distinct TF profile with high *FOSL1* and *SNAI2* activity and vimentin expression (Fig 1D, Fig S1C). The remaining SCL PDOs (PCA3, 8, 11, 12, 13, 20, 25, 27) showed more variable TF activity in *JUNB*, *FOSL2*, *TP63*, and *STAT1*, but were grouped as SCL based on shared expression of stem markers (*CD44* and *TROP2*) and their prior bulk–classification by Tang et al ^10^ (Figs 1C and 1D, Fig S1C and S1D).

As with SCL states, we examined whether NEPC PDOs exhibit additional stratification at the sc–multiome level. NEPC PDOs (PCA4, PCA10, PCA14, PCA24, LuCap49) expressed classical neuroendocrine markers, *CHGA* and *SYP*, but separated into two states: two NEPC–A PDOs (PCA14 and PCA24) showed ASCL1 expression and activity only, whereas three NEPC–A/N PDOs (PCA4, PCA10, and LuCap49) showed co–expression and activity of ASCL1 and NEUROD1 within the same cells, with lower levels of ASCL1 expression than NEPC–A (Fig 1E).

These sc–multiome defined segregation prompted us to assess whether inferred regulatory programs are reflected at the protein level. Immunofluorescence for neuroendocrine markers (*NCAM1, ASCL1, DLL3,* and *NEUROD1*) (Fig. 1F, Fig. S2) revealed substantial intra–model heterogeneity. In NEPC–A/N PDOs, ASCL1 and NEUROD1 protein levels varied substantially: a subpopulation of PCA10 cells co–expressed ASCL1 and NEUROD1, while LuCaP49 was almost uniformly ASCL1 positive. PCA14 (NEPC–A) was initially dominated by ASCL1+ cells with rare NEUROD1+ cells, but later passages showed an increased NEUROD1+ fraction, suggesting clonal drift (Fig. S2). Interestingly, ASCL1 and NEUROD1 protein distributions were not fully concordant with gene expression and chromatin accessibility, indicating additional regulatory complexity. Other lineage states (AR, TROP2, YAP/TAZ) showed expected marker patterns with occasional admixture in PDOs with mixed subpopulations (Fig S2).

We were motivated to test whether these 7 PDO–defined lineage states are representative of lineage states in advanced human prostate cancer. Using a nearest template prediction (NTP) approach with permutation–based significance testing, we analyzed single–cell RNA–seq data of metastatic CRPC from heavily–treated patients^2^ (N=14) and identified all major PDO–defined lineage states at the tumoral level (Fig 1G, Fig S1F). To account for potential sampling biases in this single cell cohort, we applied the same subtype signatures to bulk transcriptomic data from the much larger (N=429) Stand Up To Cancer (SU2C) CRPC cohort to estimate subtype composition^17^. Among the classified SU2C tumors, the AR–high subtype was the most frequent (>30%), followed by SCL/VIM+ (~20%), SCL (~10%), and NEPC–A and NEPC–A/N (~20%), whereas SCL/ASCL1–low (~8%) and WNT (~2–3%) are rarer (Fig 1G, Fig S1G). Approximately ~8–9% remain unclassified, likely reflecting lineage states not captured in the current collection of PDOs. Together, these findings establish a prostate cancer PDO resource with comprehensive single cell annotation that provides greater clarity and resolution of lineage states seen in metastatic CRPC patients.

### PDO Plasticity and Topic Modeling

**Figure 2.**
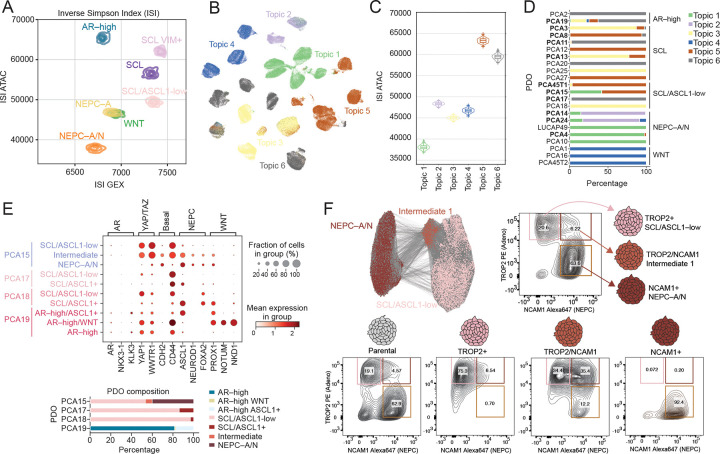
Regulatory heterogeneity and plasticity within and across prostate cancer PDOs. **(A)** Inverse Simpson Index (ISI) comparing transcriptional (GEX) and chromatin (ATAC) diversity across seven major lineage states. **(B)** Topic modeling of chromatin accessibility identifying six co–accessibility programs (Topic Modules) projected onto UMAP space. **(C)** Distribution of Inverse Simpson Index of chromatin diversity (ISI ATAC) across Topic Modules. **(D)** Fractional composition of Topic Modules within each PDOs, grouped by subtype. **(E)** Dot plot showing expression of select lineage markers across subpopulations within representative PDOs, namely PCA15, PCA17, PCA18, and PCA19 with corresponding compositional bar plots below. **(F)** UMAP showing three populations of SCL/ASCL1–low, intermediate, and NEPC–A/N found in PCA15 (left top). FACS using TROP2–PE and NCAM1–647 to identify TROP2+, NCAM1+, and double–positive intermediate populations (right). Bottom, re–plating assays of unsorted or each subpopulation, which demonstrates the lineage potential of sorted populations after 21 days of organoid culture.

While these lineage states define major transcriptional programs, our approach also sought to capture heterogeneity within each subtype and individual PDOs. Notably, several PDOs—particularly in the SCL group—lacked a dominant transcription and chromatin accessibility program. To quantify this heterogeneity, we applied the Inverse Simpson Index (ISI), a metric often used to measure ecological diversity. ISI captures either the effective number and evenness of expressed genes (ISI GEX) or accessible chromatin regions (ISI ATAC) within each PDO (Fig. S3A) or subtype while controlling for sample size (Fig 2A, Methods). Higher ISI values indicate increased regulatory diversity and lack a stable lineage commitment.

SCL, SCL/ASCL1–low, and SCL/VIM+ subtypes exhibited the highest ISI GEX and ISI ATAC scores, whereas more lineage–constrained populations––including WNT, NEPC–A/N, and NEPC–A–– showed markedly lower ISI values. These ISI scores were further supported by a CRPC patient from whom two PDOs were derived independently from two different regions of the primary prostate tumor: one with high plasticity classified as SCL/VIM+ (PCA45T1) and the second with low plasticity classified as WNT(PCA45T2). This patient subsequently developed liver metastases with NEPC histology which, based on ISI, may have arisen from the SCL/VIM+ subtype. Notably, AR–high PDOs showed low ISI GEX, consistent with an androgen–dependent adenocarcinoma phenotype, but exhibited unexpectedly high ISI ATAC (Fig 2A). This discordance, exemplified by PCA19, which harbors multiple lineage populations, is consistent with its permissive chromatin landscape. Together, these findings position ISI as a potential measure of lineage plasticity that quantitatively captures population heterogeneity, supported by orthogonal biological and clinical observations.

While ISI quantifies the extent of regulatory heterogeneity, it does not resolve the underlying programs that drive diversity. To identify the chromatin accessibility profiles of these heterogenous states, we applied unbiased topic modeling [latent Dirichlet allocation (LDA)] to the chromatin accessibility data to identify co–accessibility programs (“Topic Modules”) (Fig 2B). SCL– and AR–high populations again showed considerable variability in accessibility falling into three Topic Modules (3, 5 and 6), consistent with their high plasticity. Topics 5 and 6–– enriched in AR–high and SCL states––showed the highest ISI ATAC scores (Fig 2C). Topic 5 was enriched for *FOSL1* and other *AP1* factors, whereas Topic 6 showed higher enrichment for STAT1, potentially representing previously described JAK/STAT driven states (Fig S3B).

A complementary approach that leverages multiomics data is Spectra matrix factorization on highly variable genes (“Hotspot Modules”) (Fig S3C and S3D, and GSEA analysis shown in Fig S4A). These Hotspot modules showed substantial overlap with Topic Modules, except for Topic Module 3 and Hotspot Module 5 (Fig S4B). All TFs enriched in both Topic and Hotspot modules are in Table S2 and S3, respectively. Interestingly, Hotspot and Topic Modules failed to fully separate CRPC–AR and CRPC–SCL PDOs,^8^ suggesting that some AR–high PDOs may share regulatory networks with SCL PDOs rather than being governed by fully distinct lineage–specific programs. This limitation also raises the possibility of multiple regulatory programs co–existing within individual PDOs, with implications for intra–PDO heterogeneity.

Building on this, we next asked whether regulatory programs co–exist within individual PDOs. Analysis of Topic Module composition revealed that 13 PDOs contained mixtures of two or more modules (Fig 2D), suggesting that PDO culture conditions do not select for a single dominant clone but instead maintain co–existing cellular states over time. A similar degree of subtype mixing was seen in our cluster–based annotations (Fig S4C). Focusing on these highly admixed PDOs, PCA19 (AR–high) showed three subpopulations: predominantly AR–high, with smaller WNT and ASCL1 subpopulations. PCA17 and PCA18 (SCL/ASCL1–low) both showed a dominant HNFA+ and a minor ASCL1–high (<20%) population, whereas PCA15 (mixed SCL and ASCL1) contained nearly equal SCL and NEPC (NCAM+) subpopulations with a small intermediate/hybrid subpopulation (Figs 2E and 2F).

The differential expression of the cell surface proteins TROP2 and NCAM1 within the SCL and NEPC subpopulations respectively, coupled with a high ISI score for SCL, provides an opportunity to directly address intra–tumoral plasticity through FACS–based isolation of these subpopulations. As expected, flow cytometry using TROP2–PE and NCAM2–Alexa 647 co–staining revealed three populations: TROP2–positive, NCAM1–positive, and TROP2/NCAM1–double positive. Sorting and reseeding revealed that only the double–positive subpopulation could regenerate all three lineages after 14 days, indicating a unique bipotent population that mediated NEPC transition (Fig 2F). The TROP2–positive population did give rise to a small fraction of double–positive cells (<10%), suggesting it has some potential to revert to an intermediate state whereas the NCAM1–positive cells exclusively gave rise to a homogenously NCAM1–positive population, consistent with the NEPC ISI scores predictive of low plasticity potential. Together, these results show that multiple regulatory states co–exist across and within organoids, particularly in SCL populations, and that ATAC–based measures identify organoids poised for plasticity, as exemplified by PCA15 and PCA19.

### CRISPR Screens for Synthetic Lethality

**Figure 3.**
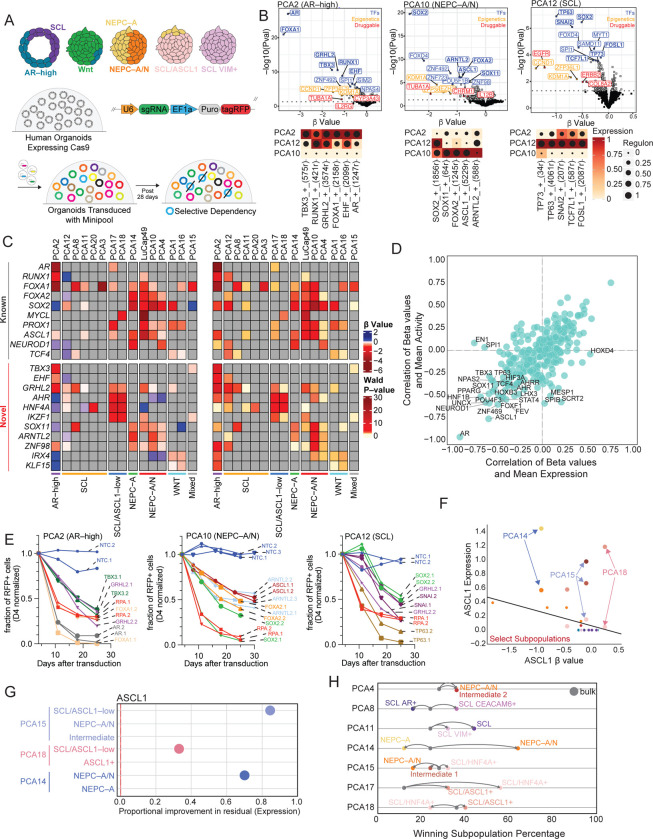
Lineage–resolved CRISPR screening identifies subtype–specific dependencies. **(A)** Schematic of pooled CRISPR–Cas9 screening in PDOs of different lineages. **(B)** Volcano plots showing gene–level depletion (β value) and significance (–log10 P value) for representative AR–high (PCA2), NEPC–A/N (PCA10), and SCL (PCA12) PDOs, with selected top 10 transcription factor, and top 3 epigenetic and druggable targets highlighted. Bolded TFs show overlap with SCENIC+ analysis for enrichment in particular PDO and shown as a heatmap below with scaled mean expression (color) and regulon activity (dot size) using SCENIC+. **(C)** Heatmaps of CRISPR dependencies across PDOs for known (top) and newly identified (bottom) transcription factor dependencies with left showing depletion value (β value) and right showing significance (–log10 P value). Only significant depleters are shown (P< 0.01). **(D)** Correlation between CRISPR depletion (β values) and gene expression or regulon activity, highlighting candidate dependencies. **(E)** sgRNA competition assays validating lineage–specific dependencies across PDOs over time with fraction of RFP shown on y–axis. Data is normalized to day 4 of transduction efficiency. **(F)** Subpopulation–resolved analysis of *ASCL1* dependency showing divergence between bulk and subpopulation–specific effects, namely PCA14, PCA15, and PCA18. **(G)** Improvement in model residuals when incorporating subpopulation–level expression for *ASCL1* across select PDOs. **(H)** Distribution of “winning” subpopulations that best explain gene–level depletion signals across TFs screens in multiple admixed PDOs.

The diversity of regulatory programs within and across PDOs raises a key question of which genetic dependencies sustain specific cell states, and how intratumoral heterogeneity may influence their detection. To address this, we performed pooled CRISPR–Cas9 dropout screens in PDOs using single guide (sgRNA) containing customized libraries (Fig 3A). Unlike conventional 2D cell line screens, CRISPR screening in 3D organoid systems presents additional challenges, including maintaining library representation and achieving sufficient sensitivity to detect fitness effects in clonally expanding structures^18^. As CRISPR screens in 3D human PDO cultures have yet to be performed extensively, we first optimized key experimental parameters to ensure robust and interpretable gene depletion results (Methods, Fig S5A–B). For this, we generated mini–CRISPR libraries targeting 843 differentially expressed genes across PDO subtypes and stratified them into three functional pools: Pool 1–TF (transcription factors), Pool 2–Epigenetics/Kinases (epigenetic regulators and kinases), and Pool 3–Druggable (druggable targets). Each pool contained four sgRNA for 281 differentially expressed genes with 5% non–targeting and SAFE control guides (Methods, Table S4).

To establish feasibility and robustness, we utilized three PDOs with divergent lineage programs and chromatin states that may pose different technical challenges: AR–high (PCA2), NEPC–A/N (PCA10), and SCL (PCA12). PDOs stably expressing Cas9 were transduced with each library, and genomic DNA was collected at baseline (T_0_) and after 21 or 28 days (T_1_ or T_2_, ~8–10 doublings). Gene level dependencies were quantified using MAGeCK based on sgRNA depletion (β value) and statistical significance (Wald P value) (Table S5). Consistent with lineage–specific dependencies for Pool 1–TF screens, the AR–high PDO (PCA2) showed strong depletion for *AR* and *FOXA1*, the NEPC–A/N PDO (PCA10) showed depletion for *SOX2*, *ASCL1* and *FOXA2*, and the SCL PDO (PCA12) was dependent on *TP63* and *SNAI2*. Notably, the top subtype–specific TF dependencies corresponded to the TF expression and regulon activity (SCENIC+) in the sc–multiome atlas (Fig 3B, Table 1, Table S5).

Pool 2–Epigenetics/Kinases and Pool 3–Druggable screens also identified subtype–specific (as well as shared) vulnerabilities. *LSD1/KDM1A* and *TUBA1A* were depleted across all three PDOs with the strongest depletion in the NEPC–A/N PDO (PCA10). *ZFP36L2* and *EZH2* were also depleted strongly in PCA10 but more modestly in the AR–high PDO (PCA2), consistent with known function in these lineage states^13, 19, 20^. Several genes stood out for their strong and unique depletion including *HNRNPCL1*, *PTHLH*, *COL4A1*, and *IL12B* in the NEPC–A/N PDO. *HKDC1* (a hexokinase) and *IL2RG* were uniquely depleted in the AR–high PDO, whereas *ZFP36L1* and *EGFR* were uniquely depleted in the SCL PDO (Fig 3B, Table S5). Overall, the mini–CRISPR screen confirmed expected lineage–specific dependencies while revealing additional subtype-specific vulnerabilities.

Given our successful optimization of small scale CRISPR screens––defined by the robust depletion of known TFs in distinct lineage states––we expanded the Pool 1–TF screen to an additional 12 PDOs with the goal of constructing a TF dependency atlas across prostate cancer lineage states. All four NEPC PDOs showed significant *ASCL1* dependence, as expected. LuCaP49 showed the strongest *ASCL1* dependence, along with dependencies on *MYCL* and *PROX1*, whereas PCA4 showed greater dependence on *NEUROD1*, together with ASCL1 dependence. Other pan–NEPC TF dependencies include *SOX2*, *FOXA1* and *FOXA2* with higher β–values overall for *FOXA2* versus *FOXA1*, consistent with the known shift from *FOXA1* to *FOXA2* expression when CRPC transitions to NEPC (Fig 3C, Table 1, Table S5).

Beyond canonical NEPC regulators, we identified three previously unreported NEPC–selective dependencies. *SOX11* sgRNAs were depleted in all NEPC PDOs. sgRNAs for *ARNTL2* (aryl hydrocarbon receptor nuclear translocator like 2), a circadian rhythm regulator that dimerizes to CLOCK protein and is implicated in advanced lung adenocarcinoma^21^, were depleted in 3 of 4 NEPC PDOs but not in other PDO subgroups. Lastly, sgRNAs for the zinc finger TF *ZNF98* were depleted across all 4 NEPC PDOs (Fig 3C, Table 1, Table S5).

In the AR–high state (PCA2), top ranking dependencies were *TBX3*, *GRHL2*, *RUNX1*, and *EHF*, in addition to well established dependencies on *AR* and *FOXA1* (Fig 3C, Table 1, Table S5). For WNT subtype PDOs (PCA1, 16), the screen revealed dependencies on *PROX1*, *IRX4* and *KLF15*, as well as known dependencies on *TCF7L1* and *TCF4* (Fig 3C, Table 1, Table S5). For SCL/ASCL1–low subtype PDOs (PCA17, 18), we identified three novel TF candidates: *AHR*, *HNF4A*, and *IKZF1*. We did not identity any consistent SCL subtype dependencies, in line with the high level of heterogeneity (ISI) seen in our sc–multiomics analyses (Fig 3C, Table 1, Table S5).

To address whether pooled functional screens could be scaled beyond the size of our mini–pooled libraries, we next screened a comprehensive TF and epigenetics factors sgRNA library comprising of 1,925 genes (Table S6) on three representative PDO models: PCA2 (AR–high), PCA12 (SCL), and PCA10 (NEPC–A/N). We first noted that depletion profiles for significantly depleted genes were highly concordant with those generated using the mini-Pool 1–TF and mini–Pool 2–Epigenetic/Kinases libraries, demonstrating reproducibility of β–values across screening platforms and confirming that library complexity can be scaled without introducing bottlenecking artifacts (Fig S5C). As expected, core essential genes, as defined by DepMap and Hart *et al* ^22^, were strongly depleted. Beyond these shared essentialities, the expanded library revealed subtype-specific dependencies not captured in the smaller pools, including *E2F3*, *TOX4*, and *SOX4* in NEPC–A/N. The *E2F3* dependency is notable because PCA10 is *RB1*–null, consistent with reduced *CCND1* dependence and increased reliance on E2F–mediated proliferation. Other top ranked dependencies include *KAT7* and *TTF1* in SCL PCA12, as well as *HOXA13,* a known regulator of luminal identity, and *ACO2* in AR–high PCA2 (Fig S5D, Table S7).

We next asked whether elevated gene expression and activity predict dependency, focusing on “dosage–sensitive” genes whose dependency is highly correlated with expression/activity, as these represent high–priority candidates for functional validation. By integrating CRISPR dependency data with TF expression and/or accessibility, we identified 23 genes with moderate to strong (anti–) correlation between expression or activity and depletion [abs(r) ≥ 0.5]. In addition to genes such as *AR* and *ASCL1*, we identified *HNF1B*, *SOX11*, *UNCX*, and *NPAS2* (Fig 3D, Table S8) as novel candidates. To validate subtype–specific and gene–dose–specific dependencies revealed from the CRISPR screens, we performed competition assays. In all cases, sgRNA competition assays recapitulated the magnitude and direction of depletion observed in pooled screens, further confirming the robustness and specificity of the identified dependencies (Fig 3E, Fig S5E, Table S9). Notably, several of these dependencies exhibited depletion effects comparable to or exceeding those observed for the canonical common essential gene *RPA3*, underscoring their functional significance. Together, these data provide a lineage–resolved map of functional dependencies across prostate cancer states, showing that while lineage identity strongly shapes functional dependencies, poorly defined states such as SCL PDOs exhibit heterogeneous and less consistent vulnerabilities.

### Effects of Intra–PDO Structure and CRISPR Depletion

Our integrated datasets further enabled us to directly assess how intra–PDO heterogeneity shapes gene–level depletion signals in pooled CRISPR screens. As depletion in heterogenous PDOs reflects the aggregate contribution of multiple subpopulations rather than a single uniform dependency, we implemented a leave–one–out modeling framework in which each PDO was excluded during model fitting, and gene expression or regulatory activity was inferred from CRISPR depletion (β values). Model performance was evaluated using the proportion of variance explained (R^2^), enabling direct comparison of bulk *versus* subpopulation–resolved predictions (Fig S6A).

*ASCL1* served as a prime example of this effect. Despite high expression across multiple NEPC PDOs, its depletion varied widely. At the bulk level, *ASCL1* expression and depletion were weakly correlated (Fig S6B). However, subpopulation–resolved analysis revealed that depletion was in fact driven by specific NEPC subpopulations (Fig 3F and Fig S6C). In NEPC PDO PCA14, replacing bulk averages with subpopulation–specific values showed that *ASCL1* depletion was primarily attributable to a NEPC–A population lacking *NEUROD1*, resulting in a substantial proportional improvement in residuals and overall model fit (Fig 3G). Similar patterns with *ASCL1* were observed in PCA15 and PCA18 (Fig 3G, Fig S6D). More broadly across other TFs, two distinct patterns were observed in PDOs where a single subpopulation accounted for most of the depletion signals (e.g. PCA14), while other depletion signals were distributed across multiple subpopulations (e.g. PCA15) (Figs S6D–G). By incorporating subpopulation–resolved modeling, *R*^2^ values for predicting key TF candidates improved compared to bulk estimates (Figs S6H). To quantify the influence of subpopulation dynamics in each PDO, we calculated the frequency with which each subpopulation emerged as the best–performing predictor (“winning subpopulation”) of gene–level depletion across all TFs included in our screens (Fig. 3H). The improved fit of subpopulation–resolved models over bulk estimates suggests that population vulnerability to TF depletion is strongly influenced by its underlying subpopulation architecture.

We then evaluated whether the expression of a given TF within a specific subpopulation determined which subpopulation predominantly drove the overall depletion. For each PDO, we applied a G–test of independence to assess whether the subpopulation providing the greatest improvement in model fit for a given TF was statistically independent of the subpopulation exhibiting the lowest expression of that TF. Low–expressing populations were disproportionately represented among the winning clones, indicating that they generally drive aggregate depletion (Table S10). Together, these results show that gene–level dependencies in heterogeneous PDOs are shaped by specific subpopulations, with resistant or low–expression populations disproportionately influencing CRISPR depletion outcomes, providing a framework to resolve lineage–specific vulnerabilities within complex PDO systems.

### AHR as a target for SCL and SCL/ASLC1 PDOs

**Figure 4.**
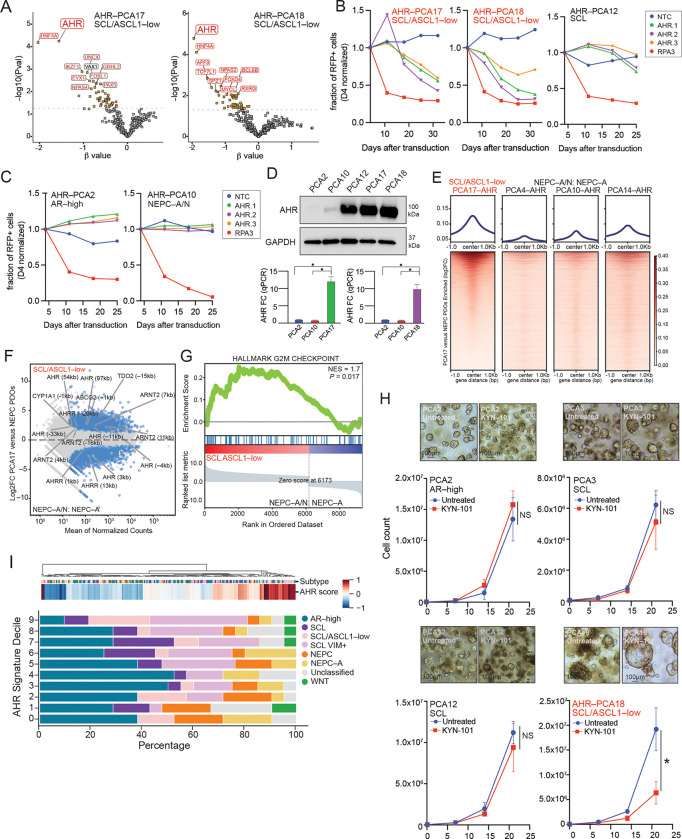
AHR is a lineage–specific dependency in SCL/ASCL1–low and SCL/VIM+ prostate cancer. **(A)** Volcano plots showing AHR as a selectively depleted gene in SCL/ASCL1–low PDOs (PCA17, PCA18). **(B)** sgRNA competition assays validating AHR dependency in SCL/ASCL1–low, PCA17 and PCA18, and more modestly in SCL PDOs, PCA12. Shown is fraction of RFP–positive cells, normalized to D4 (transduction efficiency). **(C)** AHR knockout effects across AR–high and NEPC PDOs showing increased growth or non–effect, respectively. Shown is fraction of RFP–positive cells, normalized to D4 (transduction efficiency). **(D)** Immunoblot and qPCR analysis confirming elevated AHR expression in SCL/ASCL1–low PDOs. **(E)** Volcano plot showing AHR ChIP–seq signal increased in SCL/ASCL1–low PCA17 *versus* NEPC PDOs PCA4, 10, and 14. **(F)** Differential expression of AHR target genes when analyzing AHR CHIP–seq comparing SCL/ASCL1–low and NEPC states. **(G)** Gene set enrichment analysis showing enrichment of G2/M checkpoint pathways in SCL/ASCL1–low PDOs *versus* NEPC. **(H)** Pharmacologic inhibition of AHR (KYN–101 at 1μM) across PDOs showing selective growth suppression in SCL/ASCL1–low model PCA18 with morphologic changes, but not AR–high PDOs. **(I)** Distribution of AHR signature scores across SU2C prostate cancer samples, stratified by lineage subtype showing enrichment of specific SCL subgroups in top decile of AHR scores.

Our sub–population–resolved framework revealed identification of lineage–restricted dependencies that are partially obscured in bulk analyses. Among these, AHR emerged as a top candidate selective vulnerability in SCL/ASCL1–low PDOs (PCA17 and PCA18) in our pooled screens (Fig 4A), with a more modest effect observed in the SCL organoid PCA12 (refer to Fig 3C). We therefore sought to determine whether AHR represents a functional and pharmacologically actionable target across PDO subtypes.

Using three independent AHR sgRNAs, we validated AHR dependency in competition assays in SCL/ASCL1–low subtypes PDO (PCA17, 18), observing depletion by ~17–18 days, with a more modest effect in SCL subtype PDO PCA12 (Fig 4B). In contrast, AHR knockout conferred a proliferative advantage in the AR–high PDO PCA2 but had no effect in NEPC–A/N subtype PDO PCA10 (Fig 4C) ––findings supporting a context–dependent role for AHR across lineage states.

To determine whether these differential dependencies reflect lineage–specific pathway activity, we interrogated AHR signaling across the multiome atlas. AHR expression was highest in SCL/ASCL1–low, SCL–VIM+, and SCL subtype PDOs, together with CYP1B1, a canonical AHR target gene, whereas expression of its obligate dimerization partner ARNT was relatively uniform across states (Fig S7A). These findings were independently validated by qPCR and immunoblotting, confirming that SCL/ASCL1–low PDOs express high levels of AHR (mRNA and protein) relative to AR–high and NEPC PDOs (Fig 4D).

Having confirmed differential AHR expression, we next asked whether this correlated with chromatin occupancy. AHR ChIP–seq revealed increased DNA binding in the SCL/ASCL1–low PDO PCA17 compared to three NEPC subtype PDOs (PCA10, PCA14, PCA4) (Fig 4E, Fig S7B and S7C). Furthermore, the AHR peaks in PCA17 mapped to canonical AHR target genes (*AHRR*, *CYP1A1*, *CYP2S1*, UGT1A family members, and *ABCG2*) and, through GSEA analysis, showed enrichment of AHR signaling and, importantly, G2/M checkpoint pathways (Fig 4F–G) (Table S11 and S12).

Having implicated AHR as a lineage–specific dependency within a CRPC SCL subtype, we next asked whether pharmacological inhibition of AHR could reproduce the dependency seen with CRISPR depletion. To explore this possibility, we treated 4 PDOs representing different lineage states with KYN–101, a small molecule antagonist that blocks ligand–induced AHR activation. As expected, expression of the target gene CYP1A1 was reduced following KYN–101 treatment (1μM) (Fig S7D). Consistent with the CRISPR data, KYN–101 treatment significantly impaired growth in the SCL/ASCL1–low PDO PCA18 but not in the AR–high PDO PCA2 (of note, as with the CRISPR knockout, PCA2 cells treated with KYN-101 showed increased growth, *albeit* not statistically significant) (Fig. 4H, Fig S7E).

To define the clinical context in which AHR–high prostate tumors might be targeted, we queried RNA–seq data from the SU2C cohort and identified a subset of CRPC patients with high AHR signature scores (Table S13). Furthermore, and strikingly, the top decile of AHR signature–positive patients were enriched for SCL/ASCL1–low or SCL/VIM+ lineage states, suggesting that AHR activity supports maintenance of these SCL lineages (Fig. 4I). Collectively, these findings demonstrate that AHR activity is preferentially enriched in a subset of SCL states, where it supports lineage maintenance and cellular fitness, highlighting the importance of cellular context in determining both genetic dependency and therapeutic response.

## DISCUSSION

**Figure 5.**
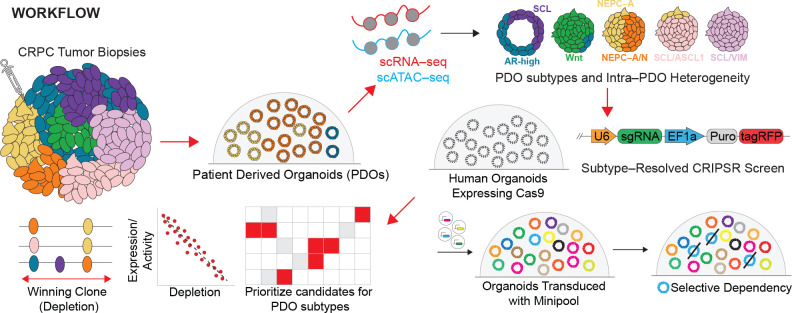
Workflow and major conclusions from integrated single–cell multiomics and CRISPR screening of heterogeneous, lineage plastic patient–derived prostate cancer organoids.

Tumor heterogeneity and lineage plasticity represent fundamental barriers to durable therapeutic control broadly across advanced solid tumors. While transcriptional and epigenetic profiling has increasingly resolved the diversity of tumor cell states, translating these descriptive atlases into actionable therapeutic strategies has remained challenging^2, 23, 24^. A major obstacle has been the lack of experimental systems that simultaneously preserve patient–relevant heterogeneity and enable scalable functional interrogation. Prostate cancer, in particular, remains underrepresented in large–scale functional genomics resources such as DepMap. Here, we address this gap by integrating single–cell multiomics profiling with pooled CRISPR–Cas9 screening across a large panel of prostate cancer PDOs to link cell state identity, regulatory programs, and functional dependencies within heterogenous human tumor models. Together, this integrated PDO atlas and screening platform provides a scalable, patient–relevant resource to define functional dependencies in advanced prostate cancer (Fig. 5).

A central conceptual advance of our work is the demonstration that tumoral heterogeneity fundamentally shapes the interpretation and predictive power of pooled CRISPR screens. Conventional functional genomics screens have largely relied on relatively homogeneous cancer cell lines representative of different lineages, which implicitly assume uniform dependency landscapes^5, 25^. Our results reveal that screens performed in PDOs, which capture the heterogeneity typically seen in patients, produce gene–level depletion signals reflect the composite of multiple subpopulations with distinct transcriptional and chromatin states. Critically, we find that the subpopulation with the lowest dependency on a given gene—the “limiting subpopulation”—disproportionately determines aggregate depletion signals, presumably because this resistant population can expand and mask vulnerabilities present in other subpopulations.

This principle has two important corollaries. First, strong lineage–restricted dependencies may appear attenuated—or even undetectable—when averaged across mixed populations, as exemplified by PCA15, where extensive admixture of SCL, neuroendocrine, and intermediate states resulted in minimal depletion of most lineage–specific regulators. Second, robust depletion of genes with low mean expression can reflect vulnerabilities confined to a functionally critical subpopulation that nonetheless limits overall population fitness; strong depletion of *HNF4A* and *AHR* in PCA17, for instance, was driven by a restricted SCL/ASCL1–low population despite low bulk expression. These observations provide a mechanistic explanation for the poor predictive performance often observed when dependencies identified in cell lines are extrapolated to patient tumors and suggest that therapeutic target selection should prioritize the activity of relevant cell states rather than population averages.

Beyond resolving technical limitations of pooled screening, our work provides new biological insight into the structure of prostate cancer plasticity. By constructing a single cell multiome atlas spanning 22 PDOs, we delineated seven major lineage states. Most notable was the refined subtyping of the previously bulk–seq–defined SCL subgroup (through bulk analyses), from which we defined three distinct cell states: SCL, SCL/ASCL1–low, and SCL/VIM+, along with the heterogeneity of NEPC (NEPC–A, NEPC–A/N), pertaining to ASCL1 or NEUROD1 expression and activity^26, 27^. Importantly, we identified intermediate and hybrid populations––often masked in bulk analyses––that exhibited a high level of diversity in chromatin accessibility and transcriptional plasticity. Quantitative measures of heterogeneity, including inverse Simpson indices and topic modeling, revealed that SCL and related AR–low states exhibit the greatest regulatory variability, consistent with their proposed role as reservoirs of lineage plasticity^2, 26, 28^.

Functional CRISPR screening across these states uncovered both shared and PDO–specific dependencies, validating known regulators while also revealing previously unrecognized vulnerabilities. Established lineage regulators, such as *AR* and *FOXA1* in AR–high, *TCF4* in WNT, and *ASCL1* and *SOX2* in NEPC^10^, emerged as top dependencies in their respective subtypes, providing internal validation of our screening approach in 3D PDO cultures. Additional subtype–specific dependences, such as *TBX3* and *EHF* in AR–high, *HNF4A* and *AHR* in SCL/ASCL1–low, and *SOX11* and *ARNTL2* in NEPC warrant further investigation. Moreover, the variable dependency of *ASCL1* and *NEUROD1* in different NEPC PDOs suggests that subpopulation structure impacts the effect of gene depletion at the time of screening.

Among these findings, the identification of AHR as a selective dependency in the SCL/ASCL1–low state offers potential for near term therapeutic relevance. AHR has been studied primarily in the context of xenobiotic metabolism and immune regulation, with limited evidence linking it to specific prostate cancer lineage states^29, 30^. Our integrated genetic, pharmacologic, and epigenomic analyses show that AHR activity is enriched in SCL/ASCL1–low population, where it supports proliferation and lineage maintenance. Notably, this dependency is highly context–specific as AHR inhibition promotes growth in AR–high tumors, underscoring the importance of lineage state in determining therapeutic response if/when AHR–directed therapy is explored clinically. This SCL/ASCL1–low state is not well represented in existing DepMap prostate cancer cell lines, highlighting a key limitation of current *in vitro* models and emphasizing the value of PDO systems for capturing clinically relevant, yet underrepresented, lineage states.

While our study establishes a scalable and integrative framework, several limitations warrant consideration. First, although CRISPR screening in PDOs can be extended to large gene libraries, genome–scale screens remain constrained by the dominance of core essential genes in pooled depletion readouts, which can obscure context–specific dependencies. Our PDO panel also has limited representation of AR–high prostate cancer states, reflecting challenges in establishing and maintaining these models and potentially biasing dependencies toward AR–low and lineage-plastic phenotypes. Second, while PDOs preserve tumor cell–intrinsic heterogeneity, they lack immune and stromal components that influence lineage stability and therapeutic response *in vivo*. Finally, organoid passaging may alter clonal composition. Although drift was observed in specific cases (e.g., PCA14), most PDOs retained diverse lineage states, supporting preservation of patient–relevant heterogeneity.

Finally, these findings are likely to extend beyond prostate cancer, as lineage plasticity and cellular heterogeneity are common across solid tumors. Integrating single–cell multiomics with functional genomics in patient–derived models provide a general framework to resolve state– specific vulnerabilities and interpret screening results in heterogeneous systems. Accounting for heterogeneity will be essential for translating functional genomics into effective therapeutic strategies.

## Supplementary Material

Supplement 1

Supplement 2

## Figures and Tables

**TABLE 1:** 

PDO	Gene	Beta value	Wald P value	Subtype
LuCap49	*MYCL*	−4.306	3.71×10^−16^	NEPC-A/N
PCA2	*FOXA1*	−3.745	1.07×10^−21^	AR-high
PCA2	*AR*	−3.549	1.06×10^−27^	AR-high
LuCap49	*ASCL1*	−2.797	3.99×10^−07^	NEPC-A/N
PCA10	*SOX2*	−2.673	1.91×10^−20^	NEPC-A/N
PCA14	*SOX2*	−2.326	6.52×10^−06^	NEPC-A
PCA17	*HNF4A*	−2.033	6.57×10^−05^	SCL/ASCL1-low
PCA18	*AHR*	−1.948	1.63×10^−05^	SCL/ASCL1-low
PCA18	*HNF4A*	−1.868	7.74×10^−05^	SCL/ASCL1-low
PCA2	*TBX3*	−1.562	2.56×10^−11^	AR-high
PCA17	*AHR*	−1.559	5.67×10^−05^	SCL/ASCL1-low
PCA4	*SOX2*	−1.348	1.78×10^−06^	NEPC-A/N
PCA14	*FOXA2*	−1.332	1.60×10^−03^	NEPC-A
PCA2	*GRHL2*	−1.308	2.91×10^−11^	AR-high
LuCap49	*SOX2*	−1.256	1.64×10^−04^	NEPC-A/N
LuCap49	*PROX1*	−1.156	3.52×10^−04^	NEPC-A/N
PCA2	*RUNX1*	−1.098	1.44×10^−09^	AR-high
PCA18	*IKZF1*	−1.042	3.16×10^−02^	SCL/ASCL1-low
PCA17	*IKZF1*	−1.034	1.29×10^−03^	SCL/ASCL1-low
LuCap49	*FOXA2*	−1.011	2.10×10^−03^	NEPC-A/N
PCA12	*TP63*	−0.994	1.27×10^−07^	SCL
PCA14	*ARNTL2*	−0.974	8.92×10^−03^	NEPC-A
PCA10	*ARNTL2*	−0.911	7.48×10^−07^	NEPC-A/N
PCA14	*ASCL1*	−0.909	2.75×10^−03^	NEPC-A
PCA10	*FOXA2*	−0.856	8.59×10^−07^	NEPC-A/N
PCA1	*PROX1*	−0.815	1.36×10^−03^	WNT
PCA10	*ASCL1*	−0.802	1.80×10^−07^	NEPC-A/N
PCA10	*ZNF98*	−0.715	1.01×10^−05^	NEPC-A/N
PCA16	*PROX1*	−0.715	1.23×10^−02^	WNT
PCA12	*SNAI2*	−0.693	8.09×10^−07^	SCL
PCA14	*ZNF98*	−0.641	4.72×10^−02^	NEPC-A
PCA1	*TCF7L1*	−0.494	3.02×10^−02^	WNT
PCA4	*FOXA2*	−0.431	3.85×10^−02^	NEPC-A/N
PCA1	*IRX4*	−0.417	2.22×10^−02^	WNT
PCA16	*IRX4*	−0.415	1.39×10^−02^	WNT
PCA4	*ARNTL2*	−0.375	1.49×10^−02^	NEPC-A/N
PCA16	*KLF15*	−0.344	2.09×10^−02^	WNT
PCA1	*TCF4*	−0.290	1.76×10^−02^	WNT
LuCap49	*ZNF98*	−0.259	3.84×10^−02^	NEPC-A/N
PCA4	*ASCL1*	−0.186	3.32×10^−02^	NEPC-A/N
PCA4	*ZNF98*	−0.179	1.91×10^−02^	NEPC-A/N
PCA16	*TCF4*	−0.111	3.68×10^−02^	WNT

AR, androgen–receptor; NEPC, neuroendocrine prostate cancer; PDO, patient–derived organoid; SCL, stem–cell–like; WNT, WNT signaling.

## Data Availability

Next–generation sequencing data have been deposited at GEO and will be publicly available as of the date of peer–reviewed publication.
