# Supplementary material for: Dependencies in heterogeneous, lineage plastic patient–derived prostate cancer organoids revealed through integrated single–cell multiomics and CRISPR screening
Source: bioRxiv. 2026 May 8:2026.05.07.723570. Preprint. [Version 1] doi: 10.64898/2026.05.07.723570 (PMC13174631; doi:10.64898/2026.05.07.723570)
Supplement: Supplement 2 [file NIHPP2026.05.07.723570v1-supplement-2.pdf]

bioRxiv preprint doi: https://doi.org/10.64898/2026.05.07.723570; this version posted May 8, 2026. The copyright holder for this preprint (which was not certified by peer review) is the author/funder, who has granted bioRxiv a license to display the preprint in perpetuity. It is made available under aCC-BY 4.0 International license.

METHODS

SINGLE–CELL MULTIOME DATA

Data pre–processing. The FASTQ files of the single–cell multiome data were processed by sample with cellranger–arc *v.* 2.0.0, except for samples PCA13, PCA19, PCA23, and PCA27, which were processed with *v.* 2.0.2. FASTQs were aligned against the reference genome hg38. All samples were aggregated using the cellranger–arc aggr function (*v.* 2.0.2). Genomic regions of the peaks were annotated with the HOMER[1] annotatePeaks.pl function, *v.* 4.11, using the cellranger-arc refdata–cellranger–arc–GRCh38-2020–A–2.0.0 genes.gtf file.

Quality control. Cell filtering criteria were computed *per* sample and separately *per* modality, and cells were then assessed independently for the quality of gene expression and chromatin accessibility data. To address sample–to–sample variability in these metrics, we set two types of QC thresholds for each metric: permissive global thresholds and sample–wise percentile thresholds. For each metric, the 2nd and 98th percentiles were computed *per* sample as sample–wise cutoffs. An exception was the fraction of mitochondrial reads, for which the 2nd percentile cutoff was not used, as low fractions of mitochondrial reads do not present a disadvantage. A cell was deemed to pass QC in a modality if it failed no more than one of the tested QC criteria. Cells were retained when both modalities passed QC.

For single–cell gene expression (GEX) data, the number of total counts *per* cell, number of genes expressed *per* cell, and fraction of mitochondrial counts *per* cell were used as QC metrics. The following permissive global thresholds were applied in addition to the sample–wise percentile

bioRxiv preprint doi: https://doi.org/10.64898/2026.05.07.723570; this version posted May 8, 2026. The copyright holder for this preprint (which was not certified by peer review) is the author/funder, who has granted bioRxiv a license to display the preprint in perpetuity. It is made available under aCC-BY 4.0 International license.

thresholds described above: library size between 2,500 and 20,000 UMIs, number of expressed genes between 200 and 10,000, and fraction of mitochondrial reads below 40%. After initial filtering, doublets were detected using the scrublet[2] approach (*v.* 0.2.3) for gene expression data with parameters min_counts = 2, min_cells = 3, vscore_percentile = 85, n_pc = 50, expected_doublet_rate = 0.02, sim_doublet_ratio = 3, and n_neighbors = 15; cells with doublet_score > 0.14 were removed.

For single–cell chromatin (ATAC) accessibility data, the number of total counts *per* cell and number of peaks detected *per* cell were used as QC metrics. The following permissive global thresholds were used in addition to the sample–wise percentile thresholds described above: library size between 1,000 and 40,000 UMIs and number of detected peaks per cell between 500 and 20,000. In addition, we added QC criteria for fraction of reads in peaks (FrIP score), TSS enrichment score, and log unique fragments. The pycistopic[3] package *v.* 1.10.12 in Python 3.8.17 was used to compute these additional metrics, and we added three additional QC criteria: FrIP scores had to be greater than 0.4, except for PCA17, where the threshold was 0.35; TSS enrichment had to be above 4; and log unique fragments had to pass a manually curated per-sample threshold. The resulting dataset consisted of 195,180 cells with multimodal profiles. Genes were filtered by computing the number of cells expressing a gene and filtering out all genes that were expressed in fewer than 3 cells. To filter peaks, a threshold of 50 cells per peak was used.

Normalization and feature selection. For single–cell GEX, normalization was performed by a shifted log–transformation of size-scaled counts[4] using the scanpy functions pp.normalize_total(target_sum = 1e4) followed by pp.log1p. For single–cell ATAC, an analogous

bioRxiv preprint doi: https://doi.org/10.64898/2026.05.07.723570; this version posted May 8, 2026. The copyright holder for this preprint (which was not certified by peer review) is the author/funder, who has granted bioRxiv a license to display the preprint in perpetuity. It is made available under aCC-BY 4.0 International license.

CPM–style log–normalization was stored as the norm layer; a Term Frequency–Inverse Document Frequency (TF–IDF) representation was additionally computed as implemented in the muon ATAC module, using the shifted log–transformation of term frequency (TF), which has been shown to be beneficial in sparse datasets compared with using TF directly as implemented in the muon ATAC module[5].

To select highly variable genes, the scanpy function pp.compute_highly_variable was applied with flavor = ‘cellranger’ and n_top_genes = 3000. To select highly variable peaks, the scanpy function pp.compute_highly_variable was applied to the log–CPM–normalized ATAC data with parameters min_mean = 0.05 and min_disp = 0.5, resulting in 12,608 highly variable peaks. Cell cycle genes were scored to determine the cell cycle phase with the scanpy tl.score_genes_cell_cycle function per sample using the human S– and G2M–phase gene lists curated by Tirosh et al[6].

Dimensionality reduction. For dimensionality reduction, PCA with 50 PCs was computed for gene expression, and for chromatin accessibility, we computed latent semantic indexing (LSI[7]), dropping the first component and retaining the next 30 dimensions, PCA on the highly variable peaks, and linear discriminant analysis (LDA) on the binarized peak matrix (see cisTopic[8]) as potential low–dimensional representations. To compute a joint UMAP projection of the multiome data, PCA representations were derived from highly variable features per modality, and the per-modality PCA embeddings were L2–normalized and used to compute a Weighted Nearest Neighbor (WNN) graph with 30 neighbors per modality; this joint graph was then used as input to the UMAP algorithm.

bioRxiv preprint doi: https://doi.org/10.64898/2026.05.07.723570; this version posted May 8, 2026. The copyright holder for this preprint (which was not certified by peer review) is the author/funder, who has granted bioRxiv a license to display the preprint in perpetuity. It is made available under aCC-BY 4.0 International license.

Batch correction. Batch correction was not performed in the final dataset because every batch correction method tested removed differences between the different subtypes of PDOs and left cell cycle variation as the dominant source of variation in the dataset, which is a clear sign of overcorrection.

Clustering and subtype characterization. Leiden[9] clustering (leidenalg package *v.* 0.10.2) was first performed on the entire atlas to annotate broad categories. To identify subpopulations within each PDO, Leiden clustering was performed for each sample separately. For each sample, unimodal GEX and ATAC clustering were computed at resolution 1.0, and joint WNN clustering was computed at a resolution of 0.8. For the GEX clustering, gene characterization was performed with a one–vs–all t–test using the scanpy tl.rank_genes_groups function with method = 't-test_overestim_var'. For differential accessibility analysis, considerable sparsity of the data was encountered; therefore, peak counts were aggregated using a pseudobulk approach. For each cluster and sample, we randomly sampled 80% of the cells and partitioned them into pseudobulk groups of 100 cells for the broad annotation or 20 cells for the subpopulation annotation. Pseudobulk counts were normalized with shifted log(CPM+1). We then characterized differentially accessible peaks using a one–vs–all Wilcoxon rank–sum test as implemented in the Muon package function atac.tl.rank_peaks_group (method = 'wilcoxon', add_peak_type = True, add_distance = True, rank_abs = True). We then inspected the top 20 genes/peaks to assess whether a cluster represented a subpopulation. We repeated clustering and gene/peak characterization on the joint WNN clusters to label clusters. To set the subpopulation annotation in the context of the entire atlas, we performed another round of gene/peak characterization on

bioRxiv preprint doi: https://doi.org/10.64898/2026.05.07.723570; this version posted May 8, 2026. The copyright holder for this preprint (which was not certified by peer review) is the author/funder, who has granted bioRxiv a license to display the preprint in perpetuity. It is made available under aCC-BY 4.0 International license.

each subpopulation with the entire PDO atlas as reference. Subpopulations were retained only when at least one of the top 20 characteristic genes/peaks was unique to the subpopulation; otherwise, they were merged with the majority label of the sample. The final annotation reflects a balance between the global context and the detailed resolution of subpopulations *per* sample.

Heterogeneity assessment. To assess group–wise heterogeneity, we derived a feature–centric metric as follows. Gene expression and chromatin accessibility were binarized per cell (X > 0). For each group (e.g., cluster or subtype), we counted the number of cells expressing a feature or accessible at a feature, normalized these counts across all features to obtain a relative abundance p_i, and computed the inverse Simpson Index (ISI)[10] to quantify the diversity (D) of expressed genes and open peaks *per* group, respectively:

$$D = \frac{1}{\sum_{i=1}^{N} p_i^2}$$

The ISI represents the number of effective features per group, such that a higher number represents higher diversity and therefore higher heterogeneity. To control for differences in sample size, we randomly sampled 1,000 cells per group and repeated draws 100 times; we report the mean ISI across draws. The ISI was computed independently over genes (GEX) and peaks (ATAC) at three groupings: main subtype (AR–high, AR–low, NEPC, WNT), refined subtype, and *per* PDO.

Subgroup analysis of gene expression modality. To perform a feature–based subgroup analysis of the gene expression modality, we used Spectra[11] and Hotspot[12]. Briefly, we computed a Spectra model on the entire PDO atlas using the cross of PDO identity and the joint subtype annotation as

bioRxiv preprint doi: https://doi.org/10.64898/2026.05.07.723570; this version posted May 8, 2026. The copyright holder for this preprint (which was not certified by peer review) is the author/funder, who has granted bioRxiv a license to display the preprint in perpetuity. It is made available under aCC-BY 4.0 International license.

the cell type annotation and using a subset of highly variable genes (parameters lam = 0.1, delta = 0.001, kappa = None, rho = 0.001, n_top_vals = 50, overlap_threshold = 0.2, gs_num = 3, num_epochs = 10,000). We used the 'global' gene–set dictionary as our *a priori* annotation dictionary and dropped all immune–related annotations. This resulted in 185 fitted factors. We then scored the gene expression for all factors using the scanpy tl.score_genes function and derived the mean *per* cell population, retaining factors whose maximum mean score across cell populations was ≥ 2; this yielded 32 factors representing 1,132 genes. To take into account the local correlation of genes, the Hotspot *v.* 1.1.1 compute_local_correlations function was applied to the retained gene set and grouped using the create_modules function in Hotspot (parameters min_gene_threshold = 30, core_only = True, fdr_threshold = 0.05), which split the genes into 15 gene modules. We then manually aggregated the 15 Hotspot modules into 6 modules by similarity in the local correlations plot. Per–module scores were computed with the scanpy tl.score_genes function, and cells were assigned to the module with the highest score. To interpret the modules, we ran over–representation analysis with the decoupler[13] Python package *v.* 1.6.0 (Python v. 3.8.17) against the MSigDB Hallmark gene sets, with the background set to all genes retained after filtering genes present in fewer than 50 cells. Significantly enriched gene sets were filtered at FDR-adjusted $p < 0.05$.

Subgroup analysis of chromatin accessibility modality. To perform a feature–based subgroup analysis of the chromatin accessibility modality, we adapted the Latent Dirichlet Allocation (LDA[14]) workflow of the pycistopic package *v.* 1.10.12, Python 3.8.17. Due to performance limitations of the original pycistopic implementation, we transformed the binarized peak matrix into a bag–of–words representation, with each cell encoded as the list of accessible peak IDs, and

bioRxiv preprint doi: https://doi.org/10.64898/2026.05.07.723570; this version posted May 8, 2026. The copyright holder for this preprint (which was not certified by peer review) is the author/funder, who has granted bioRxiv a license to display the preprint in perpetuity. It is made available under aCC-BY 4.0 International license.

replaced the python lda backend with the gensim[15] LdaMulticore implementation. LDA is a probabilistic biclustering algorithm that groups peaks by co–accessibility to a predefined number of topics and simultaneously computes, for each cell, the probability of each topic, such that cells can be mapped to topics and peaks to topics. To select the number of topics *k*, we computed LDA models with k ranging from 2 to 24 topics and assessed each model by looking at the marginal probability distribution for all topics and topic overlap through normalized mutual information, as implemented in the normalized_mutual_info_score function of the scikit-learn[16] package *v.* 1.3.0. We determined that k = 6 was the best number of topics to represent the data.

Motif enrichment analysis. For each annotated cluster, we performed differential accessibility testing in pseudobulks as described above. Peaks were retained when log-fold-change ≥ 0.6 and Benjamini-Hochberg-corrected p-value $\leq 10^{-5}$.

For each topic, we computed the standardized topic–region probability distribution for all peaks *per* topic and used the triangle thresholding method to determine the threshold to select peaks. Briefly, the triangle thresholding method is used on histograms where the histogram peak is connected with the furthest value at the histogram tail by a line, and the distance of the histogram values to this line is maximized. The histogram value where the maximum distance is observed represents the threshold.

We then used the run_pycistarget wrapper function from the SCENIC+3 package *v.* 1.0.1.dev3+g3741a4b for motif enrichment using both the Differentially Enriched Motifs (DEM) and the cisTarget (CTX) approaches, as implemented in pycistarget v. 1.0.3.dev1+g3fde1ce. We

bioRxiv preprint doi: https://doi.org/10.64898/2026.05.07.723570; this version posted May 8, 2026. The copyright holder for this preprint (which was not certified by peer review) is the author/funder, who has granted bioRxiv a license to display the preprint in perpetuity. It is made available under aCC-BY 4.0 International license.

used the per–topic peak sets, with and without promoter regions, and the per–cluster differentially accessible peak sets at both the main–subtype and subclone resolution as the foreground set, and the set of all accessible peaks as the background set. This approach computes Cis–Regulatory Module (CRM) scores for foreground and background region sets, and the distribution of scores was compared using a Wilcoxon test. Motifs with a log–fold-change > 0.5 and a Bonferroni-corrected p-value < 0.05 were considered significant.

RE–ANALYSIS OF HUMAN TUMOR SINGLE–CELL/SU2C AND NTP

Re–analysis of human prostate single–cell RNA–seq data. We obtained pre–processed count matrices for all previously published patient samples in Zaidi et al.[17], which have been deposited on GEO under the accession ID GSE264573 and https://duos.broadinstitute.org/ (accession no. DUOS-000115)[18].

Quality Control (QC). Similar to the QC of the single–cell multiomics data, we computed the number of total counts per cell, number of genes expressed per cell, and fraction of mitochondrial counts per cell. We defined the following permissive global thresholds to filter cells: library size between 500 and 100,000 UMIs, number of expressed genes between 200 and 12,000, and fraction of mitochondrial reads below 20%. In the second step, we computed the 2nd and 98th percentiles per sample for total counts per cell, number of genes expressed per cell, and fraction of mitochondrial counts to account for sample–to–sample variability in data quality. An exception was the fraction of mitochondrial reads, for which we did not use the 2nd percentile, as low fractions of mitochondrial reads do not present a disadvantage. Cells that passed all or all but one of the QC criteria were retained. After initial filtering, we detected doublets using the scrublet[2]

bioRxiv preprint doi: https://doi.org/10.64898/2026.05.07.723570; this version posted May 8, 2026. The copyright holder for this preprint (which was not certified by peer review) is the author/funder, who has granted bioRxiv a license to display the preprint in perpetuity. It is made available under aCC-BY 4.0 International license.

approach (v. 0.2.3) for gene expression data with parameters min_counts = 2, min_cells = 3, vscore_percentile = 85, n_pc = 50, expected_doublet_rate = 0.02, sim_doublet_ratio = 3, and n_neighbors = 15. The dataset after initial filtering consisted of 137,087 cells, which encompassed a mixture of tumor, stromal, and immune cells.

Normalization, feature selection and dimensionality reduction. We normalized the count matrix with shifted log–transformation of size-scaled counts. To select highly variable genes, we used the scanpy function pp.compute_highly_variable with parameters flavor = 'cellranger' and n_top_genes = 3000. We scored cell cycle genes to determine the cell cycle phase with the scanpy tl.score_genes_cell_cycle function per sample. As reference for cell cycle scoring, we used the human cell cycle gene list curated by Tirosh et al. For dimensionality reduction, we computed PCA with 20 components on the highly variable gene set using the scanpy pp.pca function and used this low-dimensional representation of the data to compute a k–nearest neighbor graph with k = 15 neighbors using the scanpy pp.neighbors function and a UMAP representation of the data using the scanpy tl.umap function.

Clustering and cell type annotation. We performed Leiden[9] clustering (leidenalg package *v.* 0.10.2) at resolution 0.5 with the scanpy tl.leiden function and computed the top 30 characteristic genes using the scanpy tl.rank_genes_groups function. We then examined these top genes per cluster and assigned clusters to broad compartments using marker genes for stroma (*COL1A2, ACTA2, PECAM1*), epithelial cells (*EPCAM*), neuroendocrine tumors (*SYP, CHGA, CHGB, ASCL1, NEUROD1*), and immune cells (pan–immune marker *PTPRC*). Within immune cells, we additionally distinguished T cells (*CD3D, IL7R, CD4, CD8A, CD69, CD38, LEF1, CCR7, TCF7, FOXP3*), NK cells (*GNLY, NKG7, KLRD1*), dendritic cells (*FCER1A, CST3*), B cells (*MS4A1,*

bioRxiv preprint doi: https://doi.org/10.64898/2026.05.07.723570; this version posted May 8, 2026. The copyright holder for this preprint (which was not certified by peer review) is the author/funder, who has granted bioRxiv a license to display the preprint in perpetuity. It is made available under aCC-BY 4.0 International license.

CD79A), plasmablasts (*MZB1, HSP90B1, FNDC3B, PRDM1, IGKC, JCHAIN*), monocytes (*CD14, LYZ, FCGR3A, MS4A7*), and erythroid cells (*HBB, HBA2, HBA1*). Clusters without expression of known markers and with inconclusive characteristic gene expression were labeled as “unknown.”

Identification of tumor cells. To distinguish tumor cells from other epithelial and stromal cells, we predicted copy number variation (CNVs) using the Python implementation of inferCNV in the infercnvpy package *v.* 0.4.5 (https://infercnvpy.readthedocs.io/en/latest/) with reference categories Monocyte, T cell, B cell, Plasmablast, and Erythroid cells. Classification into normal and tumor tissue resulted in 33,379 tumor cells, which we used for subsequent analysis.

To identify tumor subsets, we recomputed the PCA and UMAP embedding of the data, repeated Leiden clustering at resolution 0.5, and computed the top 30 characteristic genes. We further examined the gene expression of tumor, immune, and myeloid marker genes as previously described in Zaidi et al.[17] (*AR, KLK3, FOLH1, ALB, ASCL1, NEUROD1, PTPRC, LYZ, IGLC3*). In accordance with the previously described analysis, we identified an *ALB*–expressing population, which is known to be expressed in hepatocytes. We further excluded two clusters with residual *LYZ* expression, known to be expressed in monocytes, and an *IGLC3* (Immunoglobulin Lambda Constant 3)–expressing cluster, indicative of B cell lineage cells. Lastly, we identified a cluster with cells from several patients that showed comparatively low library size and number of expressed genes. The resulting tumor dataset consisted of 30,105 cells from 11 patients (patient IDs HP7, HP8, HP10, HP11, HP13, HP14, HP17, HP18, HP19, HP20, and HP21).

Tumor subset classification. We first scaled the dataset by z–score and then used the NTP algorithm (see below) to classify cells by gene signatures using the top 500 differentially expressed

bioRxiv preprint doi: https://doi.org/10.64898/2026.05.07.723570; this version posted May 8, 2026. The copyright holder for this preprint (which was not certified by peer review) is the author/funder, who has granted bioRxiv a license to display the preprint in perpetuity. It is made available under aCC-BY 4.0 International license.

genes of each major cell type lineage identified in the PDO atlas, using n_perms = 1000, BH FDR pooled across all comparisons, and assignment thresholds p_thresh = 0.05 and fdr_thresh = 0.5.

Re–Analysis of SU2C PRAD Cohort. The SU2C PRAD cohort bulk RNAseq dataset[19] was downloaded from cBioPortal (https://www.cbioportal.org/study/summary?id=prad_su2c_2019) as FPKM–normalized expression. Values were z–scored gene–wise and classified with NTP using templates built from the top 500 differentially expressed genes of each major cell–type lineage identified in the PDO atlas, as described above.

SU2C Bulk RNAseq data processing. Raw sequencing reads were demultiplexed with bcl2fastq. Reads were trimmed with cutadapt *v.* 2.621 with parameters -j 8 -e 0.1 -q 20 -O 1 and aligned to build GRCm38 of the human genome using STAR v2.7.6a22[20] with extra parameters --twopassMode Basic --outSAMunmapped Within --outSAMattributes NH HI AS NM MD --outSAMstrandField intronMotif. Alignments were quantified using all features in the Gencode version v29 M34 GTF23 with featureCounts from subread *v.* 1.6.224 in paired-end mode with options -O –fraction. Genes were then filtered down to those with at least 20 total counts across all samples across experiments.

Classification of samples using gene signatures with the Nearest Template Prediction (NTP) algorithm. The classification of human tumor samples or single cells into molecular subtypes was performed using the Nearest Template Prediction (NTP) algorithm[21, 22]. This method allows for the prediction of classes in individual samples using a predefined set of marker genes (templates) without the need for a larger training cohort. We derived the gene signatures from the major

bioRxiv preprint doi: https://doi.org/10.64898/2026.05.07.723570; this version posted May 8, 2026. The copyright holder for this preprint (which was not certified by peer review) is the author/funder, who has granted bioRxiv a license to display the preprint in perpetuity. It is made available under aCC-BY 4.0 International license.

lineages of the PDO atlas and used them to classify the SU2C PRAD cohort data[19] and the human prostate cancer tumor biopsy single-cell RNAseq data[17].

The algorithm proceeds in three stages:

1. **Template Construction:** A signature template is defined for each subtype using the top N differentially expressed genes identified from prior subcluster analysis. For each subtype, a binary template vector is created where genes specific to the subtype are assigned a value of 1, and all others are assigned 0.
2. **Distance Calculation:** For each sample, the proximity to each subtype template is evaluated. The algorithm calculates the Cosine distance between the sample's actual expression profile and the ideal template vectors.
3. **Statistical Significance:** To ensure the robustness of the assignment, a permutation test is performed. A null distribution is generated by randomly resampling gene sets to calculate the probability (p-value) of obtaining the observed proximity by chance. False Discovery Rate (FDR) correction (Benjamini-Hochberg) is applied to account for multiple testing.

Samples were assigned to the subtype with the highest proximity, provided they met the predefined significance thresholds (e.g., $p < 0.1$ and $FDR < 0.5$). Samples failing to meet these criteria were labeled as "unclassified".

We re-implemented the algorithm for compatibility with anndata v. 0.10.7, scanpy v. 1.10.0rc2.dev53+gefcf8df4, pandas v. 2.2.2, numpy v. 1.26.4 and statsmodels v.0.14.1 using Python v. 3.11.9.

bioRxiv preprint doi: https://doi.org/10.64898/2026.05.07.723570; this version posted May 8, 2026. The copyright holder for this preprint (which was not certified by peer review) is the author/funder, who has granted bioRxiv a license to display the preprint in perpetuity. It is made available under aCC-BY 4.0 International license.

AHR CHIP–SEQ DATA

Chromatin Immunoprecipitation (ChIP) and sequencing. Prostate cancer cells (~4 million per condition) were provided to the MSKCC Epigenetics Research Innovation Lab for processing. Cells were fixed with 1% formaldehyde for 10 minutes, after which the reaction was quenched by the addition of glycine to a final concentration of 0.125 M. Fixed cells were washed twice with PBS, and cell pellets were resuspended in SDS buffer (100 mM NaCl, 50 mM Tris-HCl pH 8.0, 5 mM EDTA, 0.5% SDS, 1× protease inhibitor cocktail from Roche).

The resulting nuclei were pelleted and resuspended in immunoprecipitation buffer at 1 mL per 0.5 million cells. The immunoprecipitation buffer consisted of SDS buffer and Triton dilution buffer (100 mM NaCl, 100 mM Tris-HCl pH 8.0, 5 mM EDTA, 5% Triton X-100) mixed in a 2:1 ratio, supplemented with 1× protease inhibitor cocktail (Millipore Sigma, #11836170001). Samples were processed on a Covaris LE220+ focused ultrasonicator to achieve an average chromatin fragment length of 200–300 bp using the following parameters: PIP = 420, duty factor = 30, cycles per burst = 200, time = 35 minutes. Chromatin concentrations were estimated using the Pierce™ BCA Protein Assay Kit (Thermo Fisher Scientific, #23227) according to the manufacturer's instructions. Immunoprecipitation reactions were set up in 1 mL of immunoprecipitation buffer in Protein LoBind tubes (Eppendorf, #22431081) and pre-cleared with 50 µL of Protein A/G magnetic beads (Cytiva, #9981922) for 2 hours at 4°C. After pre–clearing, samples were transferred to new Protein LoBind tubes and incubated overnight at 4°C with antibodies including AHR (rabbit monoclonal, Cell Signaling Technology, #83200) and H3K27ac (Cell Signaling Technology, #8173). The AHR antibody (CST #83200) is a rabbit monoclonal antibody recognizing the aryl hydrocarbon receptor (AHR), a ligand–activated transcription factor involved in xenobiotic metabolism and transcriptional regulation. It is validated for applications

bioRxiv preprint doi: https://doi.org/10.64898/2026.05.07.723570; this version posted May 8, 2026. The copyright holder for this preprint (which was not certified by peer review) is the author/funder, who has granted bioRxiv a license to display the preprint in perpetuity. It is made available under aCC-BY 4.0 International license.

including ChIP, Western blotting, and immunofluorescence, and is used here to detect endogenous AHR chromatin binding. Antibodies were used at a dilution of 1:50 (AHR) or 1:100 (H3K27ac).The following day, 50 µL of BSA–blocked Protein A/G magnetic beads were added to each reaction and incubated for 2 hours at 4°C. Beads were then washed twice with low-salt wash buffer (150 mM NaCl, 1% Triton X-100, 0.1% SDS, 2 mM EDTA, 20 mM Tris-HCl pH 8.0), twice with high-salt wash buffer (500 mM NaCl, 1% Triton X-100, 0.1% SDS, 2 mM EDTA, 20 mM Tris-HCl pH 8.0), twice with LiCl wash buffer (250 mM LiCl, 10 mM Tris-HCl pH 8.0, 1 mM EDTA, 1% sodium deoxycholate, 1% IGEPAL CA-630), and once with TE buffer (10 mM Tris-HCl pH 8.0, 1 mM EDTA). Samples were reverse crosslinked overnight in elution buffer (1% SDS, 0.1 M $NaHCO_3$) and purified using the ChIP DNA Clean & Concentrator kit (Zymo Research, #D5205) following the manufacturer's instructions. Recovered DNA fragments were quantified using a Qubit Flex fluorometer (Thermo Fisher Scientific) and submitted to the MSKCC Integrated Genomics Operation core facility for library preparation and sequencing. Immunoprecipitated DNA was quantified using PicoGreen, and fragment size distribution was assessed using an Agilent BioAnalyzer. Illumina sequencing libraries were prepared using the KAPA EvoPrep Kit (Roche, #10212250702) according to the manufacturer's instructions, using 0.2–5 ng input DNA and 14 cycles of PCR amplification. Barcoded libraries were sequenced on a NovaSeq X plus in a paired-end 100 bp (PE100) run using the NovaSeq Reagent Kit (200 cycles) (Illumina). An average of 40–100 million paired-end reads were generated per sample.

ChIP–Seq Analysis. ChIP sequencing reads were trimmed and filtered for quality and adapter content using version 0.6.10 of TrimGalore (https://www.bioinformatics.babraham.ac.uk/projects/trim_galore), with a quality setting of 15,

bioRxiv preprint doi: https://doi.org/10.64898/2026.05.07.723570; this version posted May 8, 2026. The copyright holder for this preprint (which was not certified by peer review) is the author/funder, who has granted bioRxiv a license to display the preprint in perpetuity. It is made available under aCC-BY 4.0 International license.

and running version 5.1 of cutadapt (https://cutadapt.readthedocs.io/en/stable/) and version 0.12.1 of FastQC (https://www.bioinformatics.babraham.ac.uk/projects/fastqc/). Reads were aligned to human assembly hg38 modified by 10X Genomics CellRanger software (version 2020-A) with bowtie2 (http://bowtie-bio.sourceforge.net/bowtie2/index.shtml) with parameters --local --very-sensitive --no-mixed --no-discordant --minins 10 --maxins 700 --dovetail and were deduplicated using MarkDuplicates in version 3.4.0 of Picard Tools (https://broadinstitute.github.io/picard/). To ascertain regions of chromatin accessibility, MACS3 version 3.0.3 (https://github.com/taoliu/MACS) was used with a q–value setting of 0.05 and scored against matched input as the control. The BEDTools suite version 2.31.1 (http://bedtools.readthedocs.io) was used to create normalized read density profiles. A global peak atlas was created by first removing blacklisted regions then merging all overlapping peaks and counting reads with version 2.1.1 of featureCounts. PyDESeq2[23] was used to normalize read density (median ratio method) and to calculate differential enrichment for all pairwise contrasts. Peak–gene associations were computed by HOMER annotatePeaks.pl function version 4.11 with the cellranger-arc refdata-cellranger-arc-GRCh38-2020-A-2.0.0 genes.gtf file. Composite and tornado plots were created using deepTools v3.5.6[24] by running computeMatrix reference-point and plotHeatmap on normalized bigwigs with flanking regions defined by the surrounding 1 kb from the peak center.

bioRxiv preprint doi: https://doi.org/10.64898/2026.05.07.723570; this version posted May 8, 2026. The copyright holder for this preprint (which was not certified by peer review) is the author/funder, who has granted bioRxiv a license to display the preprint in perpetuity. It is made available under aCC-BY 4.0 International license.

ORGANOID CULTURE AND CRISPR SCREENS

PDOs and culture. As described in Tang et al[25], metastatic prostate cancer biopsies were collected with patient informed consent being obtained prior to tissue acquisition (Memorial Sloan Kettering Cancer Center, IRB 90–040, 06–107, and 16–865). Seventeen out of the 22 were previously published[25]. Patient–derived organoids (PDOs) were established and cultured as previously described[25]. Organoids were passaged every 4–7 days based on growth rates by enzymatic dissociation using TrypLE (Gibco) at 37 °C until a single–cell suspension was obtained. Digestion was supplemented with Y-27632 (10 µM) to inhibit anoikis. All lines were mycoplasma free by PCR testing.

PDO H&E and Immunofluorescence. Haematoxylin and eosin (H&E) staining was performed as previously described. Automated multiplex immunofluorescence (IF) staining was conducted using the Leica Bond BX staining system. Paraffin–embedded tissues were sectioned at 5 µm and baked at 58 °C for 1 h. Slides were loaded onto the Leica Bond platform, where IF staining was performed as follows: samples were dewaxed at 72 °C and pretreated with EDTA–based epitope retrieval ER2 solution (Leica, AR9640) for 20 min at 100 °C. Triple or quadruple antibody staining and detection were performed sequentially. Primary antibodies were incubated for 1 h at room temperature, followed by incubation with Leica Bond Polymer anti–rabbit HRP secondary antibody (included in the Polymer Refine Detection Kit, Leica, DS9800) for 8 min at room temperature. For mouse primary antibodies, a rabbit anti–mouse linker (Leica Bond Post-Primary reagent, included in the Polymer Refine Detection Kit) was applied for 8 min before incubation with the anti-rabbit HRP polymer. Signal detection was performed using Alexa Fluor tyramide signal amplification reagents (Thermo Fisher Scientific, B40953, B40958) or CF® dye tyramide

bioRxiv preprint doi: https://doi.org/10.64898/2026.05.07.723570; this version posted May 8, 2026. The copyright holder for this preprint (which was not certified by peer review) is the author/funder, who has granted bioRxiv a license to display the preprint in perpetuity. It is made available under aCC-BY 4.0 International license.

conjugates (Biotium, 92174). Complete antibody information is provided in **Supplementary Table 14.** After each round of staining, epitope retrieval was repeated to denature primary and secondary antibodies before application of the next primary antibody. Upon completion of staining, slides were washed in PBS and incubated with 4′,6–diamidino–2–phenylindole (DAPI; 5 µg/ml; Sigma-Aldrich) for 5 min, rinsed in PBS, and mounted using Mowiol 4–88 (Calbiochem). Slides were stored overnight at −20 °C before imaging.

PDO Clonality. Organoid culture and seeding experiments were performed as previously described[18, 25]. During seeding assays, organoid medium was supplemented with the anoikis inhibitor Y-27632 (10 µM). For outgrowth quantification, 100 cells were seeded per well in triplicate per condition, and organoids were counted 7 days post–seeding as three independent replicates.

PDO Cas9. Patient–derived organoids (PDOs) were transduced with Cas9 cDNA and maintained under blasticidin selection (5–15µg/mL).

Mini–pool creation RNA-seq and Differential Expression Analysis. RNA sequencing data from PDOs were analyzed in R (v4) using the DESeq2 package. Raw gene–level counts were imported, and samples were assigned to the prior bulk–derived conditions from Tang et al.: AR, NEPC, WNT, and SCL. Genes with total counts of 20 or fewer across all samples were excluded prior to analysis. Count data were normalized using variance–stabilizing transformation (VST). Differential expression between conditions was assessed using the Wald test. P values were adjusted for multiple testing using the Benjamini–Hochberg method. Genes with an adjusted P

bioRxiv preprint doi: https://doi.org/10.64898/2026.05.07.723570; this version posted May 8, 2026. The copyright holder for this preprint (which was not certified by peer review) is the author/funder, who has granted bioRxiv a license to display the preprint in perpetuity. It is made available under aCC-BY 4.0 International license.

value < 0.05 and absolute log2 fold change > 1.5 were considered significantly differentially expressed. For more permissive threshold for TFs at lower expression, we applied a threshold of log2FC >= 0.43, FDR <0.05.

Methods for sgRNA library design and cloning. sgRNA sequences were identified using CRISPick (https://portals.broadinstitute.org/gppx/crispick/public), selecting the top four sgRNAs per gene based on Pick Order ranking criteria. sgRNA sequences were divided into three sub–pools, and sgRNA oligonucleotides were synthesized on–chip (Agilent) as previously described[26]. Synthesized oligos were PCR–amplified, and amplicons were cloned via Golden Gate assembly into BsmBI restriction sites of the pUSEPR backbone[27]. Cloned libraries were amplified by electroporation into electrocompetent cells (Lucigen), and plasmid DNA was isolated using Qiagen kits. Library representation was assessed by next–generation sequencing (Illumina). List of sgRNA and/or library QC HiSeq metrics are shown in **Supplementary Table 4 and Table 6**.

Lentiviral production. Lentiviruses were produced by co–transfection of HEK293T cells (ATCC) with lentiviral backbone constructs and packaging vectors (psPAX2 and pMD2.G; Addgene 12260 and 12259) using TransIT–LT1 (Mirus Bio, MR 2306). Virus was concentrated using Lenti–X concentrator from Takara Bio.

Infection of Minipools Library 1–3 and Comprehensive Libraries. All transductions were performed by spin–infection at 1,500 r.p.m. for 1.5–2 h at room temperature. For each patient-derived organoid (PDO), cells were expanded to maintain a coverage of 8,000×. Cells were spin–infected with each given library at a multiplicity of infection (MOI) of 0.3–0.5 in 6–well plates

bioRxiv preprint doi: https://doi.org/10.64898/2026.05.07.723570; this version posted May 8, 2026. The copyright holder for this preprint (which was not certified by peer review) is the author/funder, who has granted bioRxiv a license to display the preprint in perpetuity. It is made available under aCC-BY 4.0 International license.

using viral supernatant. Viral titers for each cell line were determined by flow cytometry by calculating the percentage of TagRFP–positive cells relative to total cells. Following infection, cells were selected with puromycin for 3–4 days, after which half of the cells were harvested and frozen as the initial time point (T0), and the remaining cells were reseeded to maintain 8,000× representation. Cells were subsequently cultured and harvested at 21 and 28 days post–infection for DNA extraction.

DNA extraction and sequencing. Cell pellets were lysed, and genomic DNA was extracted using Qiagen kits and quantified by Qubit (Thermo Fisher Scientific). A quantity of genomic DNA corresponding to 5,000–8,000× representation of sgRNAs was PCR–amplified to add Illumina adapters and multiplexing barcodes. Amplicons were quantified by Qubit and Bioanalyzer (Agilent) and sequenced on an Illumina HiSeq 2500 platform. Sequencing reads were aligned to the screened library, and counts were obtained for each sgRNA.

Methods for MAGeCK analysis. FASTQ pre–processing and guide abundance were determined using the MAGeCK count command. The 5′ trim length was automatically detected by MAGeCK, and a normalized count file was generated using median normalization. Sample quality control showed greater than 85% mapped reads and a minimum sample correlation of 0.8 across all replicates. Enrichment was determined using the MAGeCK RRA (robust rank aggregation) function to obtain gene–level enrichment scores, and P values were determined by permutation. All analyses were performed using MAGeCK *v*0.5.9. All MAGeCK results are shown in **Supplementary Table 5 and Table 7**.

bioRxiv preprint doi: https://doi.org/10.64898/2026.05.07.723570; this version posted May 8, 2026. The copyright holder for this preprint (which was not certified by peer review) is the author/funder, who has granted bioRxiv a license to display the preprint in perpetuity. It is made available under aCC-BY 4.0 International license.

sgRNA depletion experiment and competition assay. Sequences for guide RNAs of interest were cloned into the pUSEPR backbone and verified by Sanger sequencing. A total of 2–4 × 10^5 cells from organoid lines stably expressing Cas9 were transduced with sgRNAs at a multiplicity of infection (MOI) of 0.3–0.4. At indicated time points, 2 × 10^5 cells were split and reseeded in 12-well plates, and the remaining cells were collected for flow cytometry analysis. Cells were imaged for TagRFP and DAPI signals at days 4, 11, 18, 25 and 32, or at days 4, 8, 16, 23, 30, 37 and 43. Data acquisition was performed using a FACSymphony A3 (BD Biosciences), and values were normalized to day 4 measurements.

PCA15 TROP2 and NCAM1 Flow Cytometry and FACS. PDO PCA15 was selected for lineage reconstitution experiments based on the presence of admixed adenocarcinoma/SCL, intermediate, and NEPC populations identified by single–cell multiome profiling. Organoids were dissociated into single cells using TrypLE Express (Gibco) supplemented with Y–27632 ROCK inhibitor (10 µM), stained for 30 minutes on ice in PBS containing 0.05% BSA and Y–27632 (10 µM) using TROP2–PE (BD Cat. 564837, Clone: 162-46, 1:100) and NCAM1–Alexa647 (BD Cat. 557711, Clone B159, 1:100). DAPI was used as a viability dye to exclude dead cells. Flow cytometric sorting was performed to isolate TROP2 single–positive (adenocarcinoma/SCL/ASCL1–low), TROP2/NCAM1 double–positive (intermediate), and NCAM1 single–positive (NEPC–A/N) populations, alongside an unsorted parental population control. Following sorting, cells were reseeded in Matrigel and cultured under standard PDO conditions for 3 weeks to assess lineage reconstitution potential. At endpoint, organoids were dissociated again into single cells using TrypLE Express supplemented with Y–27632 (10 µM),

bioRxiv preprint doi: https://doi.org/10.64898/2026.05.07.723570; this version posted May 8, 2026. The copyright holder for this preprint (which was not certified by peer review) is the author/funder, who has granted bioRxiv a license to display the preprint in perpetuity. It is made available under aCC-BY 4.0 International license.

restained with TROP2–PE and NCAM1–Alexa647, and analyzed by flow cytometry to evaluate the ability of each sorted population to regenerate different lineages.

INTEGRATION OF CRISPR AND SC–MULTIOME DATA

Subpopulation Impact Analysis. We integrated CRISPR screen results (discussed below, beta values) with single–cell multiome data aggregated at two levels of resolution: the PDO level and the subclone level. The analysis was restricted to 5 PDOs that have both pooled CRISPR screen data and a non-trivial subclone structure. For the CRISPR outcomes, we used two different significance thresholds: $p < 0.1$ for mini-libraries and $p < 0.2$ for genome-scale libraries.

Global Correlation Analysis. To identify transcription factors (TFs) whose dependencies were consistently associated with molecular states across different models, we calculated the Pearson correlation coefficient between the binarized CRISPR beta values and the mean molecular features (Expression and Activity) for each gene across all PDOs. TFs were classified as "high–correlation" if the absolute correlation coefficient exceeded 0.5.

Leave-One-Out (L1O) Linear Regression Framework. To systematically assess how well bulk molecular features predict CRISPR outcomes compared to subpopulation features, we implemented a Leave-One-Out (L1O) linear regression procedure:

- **Baseline Fit:** For each gene, a linear regression model was trained using the beta values as the independent variable to predict the molecular feature (y_{observed} = mean gene expression or activity) across all PDOs.

bioRxiv preprint doi: https://doi.org/10.64898/2026.05.07.723570; this version posted May 8, 2026. The copyright holder for this preprint (which was not certified by peer review) is the author/funder, who has granted bioRxiv a license to display the preprint in perpetuity. It is made available under aCC-BY 4.0 International license.

- **L1O Procedure:** For each specific PDO that has a subclone structure, we refit the linear model using data from all other PDOs. This "chimera" model was then used to predict the molecular feature $y$of the held–out PDO.
- **Residual Computation:** We calculated the residuals as the difference between the observed molecular feature and the predicted value (y) derived from the L1O model:

$$d(y, y_{\text{observed}}) = y_{\text{observed}} - y$$

- **Relative change in R^2:** For each gene and PDO, we computed the relative change in the coefficient of determination (R^2) when the held-out PDO or one of its subpopulations was virtually included in the regression without refitting the model:

$$\frac{R^2(y_{\text{observed}}) - R^2(y_{\text{bulkPDO}})}{R^2(y_{\text{bulkPDO}})}$$

Evaluating Subpopulation Improvement. We evaluated whether molecular data from specific subclones provided a better fit for the observed CRISPR depletion than the bulk PDO data.

- **Proportional Improvement in Residual:** We calculated the relative reduction in the absolute residual when replacing the bulk PDO value with a subpopulation–specific value:

$$p(y_{\text{subpopulation}}, y_{\text{bulkPDO}}) = \frac{\mid d(y, y_{\text{bulkPDO}}) \mid - \mid d(y, y_{\text{subpopulation}}) \mid}{\mid d(y, y_{\text{bulkPDO}}) \mid}$$

  A positive value indicates that the subpopulation molecular state more closely aligns with the linear trend established by the rest of the dataset/

- **R^2Optimization ("Winning Subpopulation" Analysis):** For each gene and PDO, we identified the "winning subpopulation" — the subpopulation population that maximized the coefficient of determination (R^2) when included in the regression. We then refit the global linear model using these optimal subpopulation features to determine the "Best R^2,"

bioRxiv preprint doi: https://doi.org/10.64898/2026.05.07.723570; this version posted May 8, 2026. The copyright holder for this preprint (which was not certified by peer review) is the author/funder, who has granted bioRxiv a license to display the preprint in perpetuity. It is made available under aCC-BY 4.0 International license.

comparing it against the "Baseline R^2" (derived from the baseline model described above) to quantify the total gain in explanatory power provided by accounting for subpopulation heterogeneity.

Statistical Independence Testing. To determine whether the improvement in model fit was driven by molecular features of the specific subpopulations, such as a subpopulation having the lowest expression, we employed a G–test, a likelihood–ratio test. This test evaluated the independence between the subpopulation with the minimum feature value and the su subpopulation bclone providing the maximum R^2 improvement across all genes and PDOs.

AHR STUDIES

KYN-101 studies. A total of 0.04×10^6 cells per well were plated with or without 1 µM KYN-101(MedChemExpress, Cat #HY–134217) in triplicate in a 24–well plate format. Cells were counted, split, and re–treated with drug every 7 days for a total duration of 21 days. Cell counts were adjusted to account for reseeding fractions used for continued culture and scaled linearly to reflect the expected cell numbers if the entire culture had been maintained.

qPCR. RNA was extracted from cells using the RNeasy Mini Kit (Qiagen) according to the manufacturer's instructions. Isolated RNA was quantified using a NanoDrop spectrophotometer and stored at −80 °C. cDNA was synthesized from 500 ng of RNA using the LunaScript RT SuperMix Kit (New England Biolabs) according to the manufacturer's protocol and stored at −20 °C. Quantitative PCR (qPCR) was performed using 1 µl of cDNA diluted 1:10, 5 µl of

bioRxiv preprint doi: https://doi.org/10.64898/2026.05.07.723570; this version posted May 8, 2026. The copyright holder for this preprint (which was not certified by peer review) is the author/funder, who has granted bioRxiv a license to display the preprint in perpetuity. It is made available under aCC-BY 4.0 International license.

SsoAdvanced Universal SYBR Green Supermix (Bio-Rad), and 0.5 µl of PrimePCR primers (Bio-Rad) in a total reaction volume of 10 µl. Primers were designed to span introns for target genes. Reactions were run in technical triplicate on a ViiA 7 Real-Time PCR System (Thermo Fisher Scientific). Gene expression changes were calculated using the $2^{-\Delta\Delta Ct}$ method.

Western blot. PDOs were treated with cell recovery solution (Corning), incubated, and washed with PBS to remove Matrigel. Cells were lysed in 1× RIPA lysis buffer (Millipore) supplemented with protease and phosphatase inhibitors, with vortexing every 3 min for a total of 30 min. Lysates were clarified by centrifugation at maximum speed for 10 min at 4 °C. Protein concentration was determined using the Pierce BCA Protein Assay Kit (Thermo Fisher Scientific). Equal amounts of protein were mixed with 1× Laemmli sample buffer (Bio-Rad) and boiled for 10 min before separation by SDS–PAGE on 4–15% Mini-PROTEAN TGX Stain-Free gels (Bio-Rad). Proteins were transferred onto PVDF membranes (Bio-Rad), and a Full Range Rainbow recombinant protein ladder (Cytiva) was used as a molecular weight marker. Membranes were blocked in 5% non-fat dry milk in 0.1% Tween-20 TBST for 1 h at room temperature with agitation and incubated overnight at 4 °C with primary antibodies diluted 1:1,000 in 5% milk. Membranes were washed three times for 15 min in 0.1% Tween-20 TBST and then incubated with secondary antibodies diluted 1:5,000 in 5% milk for 1 h at room temperature. Membranes were subsequently washed repeatedly over 30 min in 0.1% Tween-20 TBST. Signal was detected using ECL Prime Western Blotting Detection Reagent (Cytiva) and imaged on a ChemiDoc imaging system (Bio-Rad).

Data and code availability. Next–generation sequencing data have been deposited at GEO and will be publicly available as of the date of peer–reviewed publication.

bioRxiv preprint doi: https://doi.org/10.64898/2026.05.07.723570; this version posted May 8, 2026. The copyright holder for this preprint (which was not certified by peer review) is the author/funder, who has granted bioRxiv a license to display the preprint in perpetuity. It is made available under aCC-BY 4.0 International license.

Supplementary References

1. Heinz S, Benner C, Spann N, Bertolino E, Lin YC, Laslo P, Cheng JX, Murre C, Singh H, Glass CK. Simple combinations of lineage-determining transcription factors prime cis-regulatory elements required for macrophage and B cell identities. Mol Cell. 2010;38(4):576–89. doi: 10.1016/j.molcel.2010.05.004. PubMed PMID: 20513432; PMCID: PMC2898526.
2. Wolock SL, Lopez R, Klein AM. Scrublet: Computational Identification of Cell Doublets in Single-Cell Transcriptomic Data. Cell Syst. 2019;8(4):281–91 e9. Epub 20190403. doi: 10.1016/j.cels.2018.11.005. PubMed PMID: 30954476; PMCID: PMC6625319.
3. Bravo Gonzalez-Blas C, De Winter S, Hulselmans G, Hecker N, Matetovici I, Christiaens V, Poovathingal S, Wouters J, Aibar S, Aerts S. SCENIC+: single-cell multiomic inference of enhancers and gene regulatory networks. Nat Methods. 2023;20(9):1355–67. Epub 20230713. doi: 10.1038/s41592-023-01938-4. PubMed PMID: 37443338; PMCID: PMC10482700.
4. Ahlmann-Eltze C, Huber W. Comparison of transformations for single-cell RNA-seq data. Nat Methods. 2023;20(5):665–72. Epub 20230410. doi: 10.1038/s41592-023-01814-1. PubMed PMID: 37037999; PMCID: PMC10172138.
5. Bredikhin D, Kats I, Stegle O. MUON: multimodal omics analysis framework. Genome Biol. 2022;23(1):42. Epub 20220201. doi: 10.1186/s13059-021-02577-8. PubMed PMID: 35105358; PMCID: PMC8805324.
6. Tirosh I, Izar B, Prakadan SM, Wadsworth MH, 2nd, Treacy D, Trombetta JJ, Rotem A, Rodman C, Lian C, Murphy G, Fallahi-Sichani M, Dutton-Regester K, Lin JR, Cohen O, Shah P, Lu D, Genshaft AS, Hughes TK, Ziegler CG, Kazer SW, Gaillard A, Kolb KE, Villani AC, Johannessen CM, Andreev AY, Van Allen EM, Bertagnolli M, Sorger PK, Sullivan RJ, Flaherty KT, Frederick DT, Jane-Valbuena J, Yoon CH, Rozenblatt-Rosen O, Shalek AK, Regev A, Garraway LA. Dissecting the multicellular ecosystem of metastatic melanoma by single-cell RNA-seq. Science. 2016;352(6282):189–96. doi: 10.1126/science.aad0501. PubMed PMID: 27124452; PMCID: PMC4944528.
7. Cusanovich DA, Hill AJ, Aghamirzaie D, Daza RM, Pliner HA, Berletch JB, Filippova GN, Huang X, Christiansen L, DeWitt WS, Lee C, Regalado SG, Read DF, Steemers FJ, Disteche CM, Trapnell C, Shendure J. A Single-Cell Atlas of In Vivo Mammalian Chromatin Accessibility. Cell. 2018;174(5):1309–24 e18. Epub 20180802. doi: 10.1016/j.cell.2018.06.052. PubMed PMID: 30078704; PMCID: PMC6158300.
8. Bravo Gonzalez-Blas C, Minnoye L, Papasokrati D, Aibar S, Hulselmans G, Christiaens V, Davie K, Wouters J, Aerts S. cisTopic: cis-regulatory topic modeling on single-cell ATAC-seq data. Nat Methods. 2019;16(5):397–400. Epub 20190408. doi: 10.1038/s41592-019-0367-1. PubMed PMID: 30962623; PMCID: PMC6517279.
9. Traag VA, Waltman L, van Eck NJ. From Louvain to Leiden: guaranteeing well-connected communities. Sci Rep. 2019;9(1):5233. Epub 20190326. doi: 10.1038/s41598-019-41695-z. PubMed PMID: 30914743; PMCID: PMC6435756.
10. SIMPSON EH. Measurement of Diversity. Nature. 1949;163:688.
11. Kunes RZ, Walle T, Land M, Nawy T, Pe'er D. Supervised discovery of interpretable gene programs from single-cell data. Nat Biotechnol. 2024;42(7):1084–95. Epub 20230921. doi: 10.1038/s41587-023-01940-3. PubMed PMID: 37735262; PMCID: PMC10958532.
12. DeTomaso D, Yosef N. Hotspot identifies informative gene modules across modalities of single-cell genomics. Cell Syst. 2021;12(5):446–56 e9. Epub 20210504. doi: 10.1016/j.cels.2021.04.005. PubMed PMID: 33951459.

bioRxiv preprint doi: https://doi.org/10.64898/2026.05.07.723570; this version posted May 8, 2026. The copyright holder for this preprint (which was not certified by peer review) is the author/funder, who has granted bioRxiv a license to display the preprint in perpetuity. It is made available under aCC-BY 4.0 International license.

13. Badia IMP, Velez Santiago J, Braunger J, Geiss C, Dimitrov D, Muller-Dott S, Taus P, Dugourd A, Holland CH, Ramirez Flores RO, Saez-Rodriguez J. decoupleR: ensemble of computational methods to infer biological activities from omics data. Bioinform Adv. 2022;2(1):vbac016. Epub 20220308. doi: 10.1093/bioadv/vbac016. PubMed PMID: 36699385; PMCID: PMC9710656.
14. Griffiths TL, Steyvers M. Finding scientific topics. Proc Natl Acad Sci U S A. 2004;101 Suppl 1(Suppl 1):5228–35. Epub 20040210. doi: 10.1073/pnas.0307752101. PubMed PMID: 14872004; PMCID: PMC387300.
15. Řehůřek RS, P. . Software Framework for Topic Modelling with Large Corpora. Proceedings of the LREC 2010 Workshop on New Challenges for NLP Frameworks. 2010:45–50.
16. Tanaka T. [[Fundamentals] 5. Python+scikit-learn for Machine Learning in Medical Imaging]. Nihon Hoshasen Gijutsu Gakkai Zasshi. 2023;79(10):1189–93. doi: 10.6009/jjrt.2023-2266. PubMed PMID: 37866903.
17. Zaidi S, Park J, Chan JM, Roudier MP, Zhao JL, Gopalan A, Wadosky KM, Patel RA, Sayar E, Karthaus WR, Kates DH, Chaudhary O, Xu T, Masilionis I, Mazutis L, Chaligne R, Obradovic A, Linkov I, Barlas A, Jungbluth AA, Rekhtman N, Silber J, Manova-Todorova K, Watson PA, True LD, Morrissey C, Scher HI, Rathkopf DE, Morris MJ, Goodrich DW, Choi J, Nelson PS, Haffner MC, Sawyers CL. Single-cell analysis of treatment-resistant prostate cancer: Implications of cell state changes for cell surface antigen-targeted therapies. Proc Natl Acad Sci U S A. 2024;121(28):e2322203121. Epub 20240705. doi: 10.1073/pnas.2322203121. PubMed PMID: 38968122; PMCID: PMC11252802.
18. Karthaus WR, Hofree M, Choi D, Linton EL, Turkekul M, Bejnood A, Carver B, Gopalan A, Abida W, Laudone V, Biton M, Chaudhary O, Xu T, Masilionis I, Manova K, Mazutis L, Pe'er D, Regev A, Sawyers CL. Regenerative potential of prostate luminal cells revealed by single-cell analysis. Science. 2020;368(6490):497–505. doi: 10.1126/science.aay0267. PubMed PMID: 32355025; PMCID: PMC7313621.
19. Abida W, Cyrta J, Heller G, Prandi D, Armenia J, Coleman I, Cieslik M, Benelli M, Robinson D, Van Allen EM, Sboner A, Fedrizzi T, Mosquera JM, Robinson BD, De Sarkar N, Kunju LP, Tomlins S, Wu YM, Nava Rodrigues D, Loda M, Gopalan A, Reuter VE, Pritchard CC, Mateo J, Bianchini D, Miranda S, Carreira S, Rescigno P, Filipenko J, Vinson J, Montgomery RB, Beltran H, Heath EI, Scher HI, Kantoff PW, Taplin ME, Schultz N, deBono JS, Demichelis F, Nelson PS, Rubin MA, Chinnaiyan AM, Sawyers CL. Genomic correlates of clinical outcome in advanced prostate cancer. Proc Natl Acad Sci U S A. 2019;116(23):11428–36. Epub 20190506. doi: 10.1073/pnas.1902651116. PubMed PMID: 31061129; PMCID: PMC6561293.
20. Dobin A, Davis CA, Schlesinger F, Drenkow J, Zaleski C, Jha S, Batut P, Chaisson M, Gingeras TR. STAR: ultrafast universal RNA-seq aligner. Bioinformatics. 2013;29(1):15–21. Epub 20121025. doi: 10.1093/bioinformatics/bts635. PubMed PMID: 23104886; PMCID: PMC3530905.
21. Hoshida Y. Nearest template prediction: a single-sample-based flexible class prediction with confidence assessment. PLoS One. 2010;5(11):e15543. Epub 20101123. doi: 10.1371/journal.pone.0015543. PubMed PMID: 21124904; PMCID: PMC2990751.
22. Tang F, Xu D, Wang S, Wong CK, Martinez-Fundichely A, Lee CJ, Cohen S, Park J, Hill CE, Eng K, Bareja R, Han T, Liu EM, Palladino A, Di W, Gao D, Abida W, Beg S, Puca L, Meneses M, de Stanchina E, Berger MF, Gopalan A, Dow LE, Mosquera JM, Beltran H, Sternberg CN, Chi P, Scher HI, Sboner A, Chen Y, Khurana E. Chromatin profiles classify castration-

bioRxiv preprint doi: https://doi.org/10.64898/2026.05.07.723570; this version posted May 8, 2026. The copyright holder for this preprint (which was not certified by peer review) is the author/funder, who has granted bioRxiv a license to display the preprint in perpetuity. It is made available under aCC-BY 4.0 International license.

resistant prostate cancers suggesting therapeutic targets. Science. 2022;376(6596):eabe1505. Epub 20220527. doi: 10.1126/science.abe1505. PubMed PMID: 35617398; PMCID: PMC9299269.

23. Muzellec B, Telenczuk M, Cabeli V, Andreux M. PyDESeq2: a python package for bulk RNA-seq differential expression analysis. Bioinformatics. 2023;39(9). doi: 10.1093/bioinformatics/btad547. PubMed PMID: 37669147; PMCID: PMC10502239.

24. Ramirez F, Ryan DP, Gruning B, Bhardwaj V, Kilpert F, Richter AS, Heyne S, Dundar F, Manke T. deepTools2: a next generation web server for deep-sequencing data analysis. Nucleic Acids Res. 2016;44(W1):W160–5. Epub 20160413. doi: 10.1093/nar/gkw257. PubMed PMID: 27079975; PMCID: PMC4987876.

25. Chan JM, Zaidi S, Love JR, Zhao JL, Setty M, Wadosky KM, Gopalan A, Choo ZN, Persad S, Choi J, LaClair J, Lawrence KE, Chaudhary O, Xu T, Masilionis I, Linkov I, Wang S, Lee C, Barlas A, Morris MJ, Mazutis L, Chaligne R, Chen Y, Goodrich DW, Karthaus WR, Pe'er D, Sawyers CL. Lineage plasticity in prostate cancer depends on JAK/STAT inflammatory signaling. Science. 2022;377(6611):1180–91. Epub 20220818. doi: 10.1126/science.abn0478. PubMed PMID: 35981096; PMCID: PMC9653178.

26. Erazo T, Evans CM, Zakheim D, Chu EL, Refermat AY, Asgari Z, Yang X, Da Silva Ferreira M, Mehta S, Russo MV, Knezevic A, Zhang XP, Chen Z, Fennell M, Garippa R, Seshan V, de Stanchina E, Barbash O, Batlevi CL, Leslie CS, Melnick AM, Younes A, Kharas MG. TP53 mutations and RNA-binding protein MUSASHI-2 drive resistance to PRMT5-targeted therapy in B-cell lymphoma. Nat Commun. 2022;13(1):5676. Epub 20220927. doi: 10.1038/s41467-022-33137-8. PubMed PMID: 36167829; PMCID: PMC9515221.

27. Hu J, Sanchez-Rivera FJ, Wang Z, Johnson GN, Ho YJ, Ganesh K, Umeda S, Gan S, Mujal AM, Delconte RB, Hampton JP, Zhao H, Kottapalli S, de Stanchina E, Iacobuzio-Donahue CA, Pe'er D, Lowe SW, Sun JC, Massague J. STING inhibits the reactivation of dormant metastasis in lung adenocarcinoma. Nature. 2023;616(7958):806–13. Epub 20230329. doi: 10.1038/s41586-023-05880-5. PubMed PMID: 36991128; PMCID: PMC10569211.
